# Respiratory complex I‐mediated NAD ^+^ regeneration regulates cancer cell proliferation through the transcriptional and translational control of *p21* ^*Cip1*^ expression by SIRT3 and SIRT7

**DOI:** 10.1002/1878-0261.13808

**Published:** 2025-01-28

**Authors:** Masato Higurashi, Kazunori Mori, Hidetsugu Nakagawa, Momoko Uchida, Fumihiro Ishikawa, Motoko Shibanuma

**Affiliations:** ^1^ Division of Cancer Cell Biology, Department of Pharmaceutical Sciences Showa University Graduate School of Pharmacy Tokyo Japan; ^2^ Center for Biotechnology Showa University Tokyo Japan

**Keywords:** breast cancer, NAD^+^, p21^Cip1^, respiratory complex I, SIRT

## Abstract

The role of the electron transport chain (ETC) in cell proliferation control beyond its crucial function in supporting ATP generation has recently emerged. In this study, we found that, among the four ETC complexes, the complex I (CI)‐mediated NAD^+^ regeneration is important for cancer cell proliferation. In cancer cells, a decrease in CI activity by RNA interference (RNAi) against NADH:ubiquinone oxidoreductase core subunit V1 (*NDUFV1*) arrested the cell cycle at the G_1_/S phase, accompanying upregulation of *p21* ^*Cip1*^ cyclin‐dependent kinase inhibitor expression. Mechanistically, a decrease in the NAD^+^/NADH ratio downregulated SIRT3 and SIRT7 function, which suppressed *p21* ^*Cip1*^ expression at the translational and transcriptional levels, respectively, resulting in the upregulation of the antiproliferative molecule. Importantly, high expression levels of the core subunits of CI correlated with poor prognosis in patients with the hormone receptor(+)/human epidermal growth factor receptor 2(−) (HR+/HER2−) subtype of breast cancer. Therefore, NDUFV1 and SIRT3/7 have emerged as promising therapeutic targets against this breast cancer subtype.

## Graphical abstract

NAD^+^ regeneration by mitochondrial complex I NADH dehydrogenase is important for cancer cell proliferation. Specifically, NAD^+^ is necessary for the activities of NAD^+^‐dependent deacetylases SIRT3 and SIRT7, which suppress the expression of *p21* ^*Cip1*^ cyclin‐dependent kinase inhibitor, an antiproliferative molecule, at the translational and transcriptional levels, respectively. Thus, the NAD^+^–SIRT3/7–p21^Cip1^ pathway couples cellular metabolism and proliferation, supporting cancer cell proliferation.

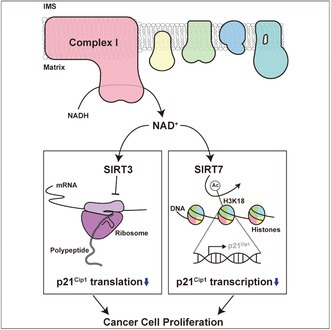

**Abbreviations**
AHA: l‐azidohomoalanine
AMA: antimycin A
ChIP: chromatin immunoprecipitation
CI: complex I
DOX: doxycycline
ETC: electron transport chain
FMN: flavin mononucleotide
H3K18Ac: acetylated lysine 18 of histone 3
HER2: human epidermal growth factor receptor 2
HR: hormone receptor
*Lb*NOX: NADH oxidase from *Lactobacillus brevis*
NDU: NADH:ubiquinone oxidoreductase core subunit V1
NMN: β‐nicotinamide mononucleotide
OXPHOS: oxidative phosphorylation
qPCR: quantitative PCR
qRT‐PCR: quantitative reverse transcription PCR
ROS: reactive oxygen species
TSA: trichostatin A

## 1 Introduction

Almost a century ago, cellular metabolism was first related to tumorigenesis by Otto Warburg, who observed anomalous metabolic characteristics of malignant tumors. This is known as the Warburg effect or aerobic glycolysis—the process fermenting glucose to lactate in the presence of ample amount of oxygen [1, 2]. From late 1990s–2000s, cellular metabolism was directly interrelated with tumorigenesis by a series of pioneering studies, which associated the deregulation of cellular metabolism in cancer cells with the activation of oncogenes (MYC, etc.) [3, 4], inactivation of tumor suppressors, including p53 [5, 6], and upregulation of signaling pathways, such as PI3K/AKT/mTOR [7, 8]. These findings enabled us to explain the metabolic characteristics of cancer cells, including their reliance on aerobic glycolysis, at least partially [1, 2]. In 2011, deregulated cellular metabolism was qualified as one of the hallmarks of cancer [9].

Subsequent studies reported that many tumors that rely on aerobic glycolysis are also reliant on mitochondrial respiration or oxidative phosphorylation (OXPHOS) [1], although it was against Warburg's interpretation that aerobic glycolysis is a consequence of irreversible mitochondrial respiratory defects [10]. Therefore, the original theory of the Warburg effect or aerobic glycolysis has been remodeled [11], and mitochondrial respiration is accepted as being indispensable for cancer cell proliferation. For example, cell proliferation is impaired by the pharmacological or genetic inhibition of the electron transport chain (ETC) [12, 13, 14, 15, 16].

ETC involves coupling nutrient oxidation to ATP generation or OXPHOS, thereby supporting cell proliferation. However, studies reported that OXPHOS is not essential for cancer cell proliferation [13, 14], suggesting that ETC has a role in supporting cancer cell proliferation beyond ATP generation. Thus, the additional roles of ETC have been recently highlighted in cell proliferation control. For example, we previously showed that reactive oxygen species (ROS) produced by ETC regulate E2F1‐mediated transcription and regulate cell cycle progression [17]. Others have highlighted the importance of ETC in the regeneration of electron acceptors [13, 14, 18]. ETC could regulate cellular senescence and autophagy [19, 20]. Thus, roles of ETC have recently expanded extensively.

In this study, we aimed to explore as yet unknown roles of ETC in cell proliferation control. Our results couple the regeneration of NAD^+^, an ETC function assumed by complex I (CI), with cell proliferation control through the regulation of SIRT activity and *p21* ^*Cip1*^ expression. Database analyses have suggested that CI functionality is related to poor prognosis in patients with cancer, specifically with the hormone receptor (HR)(+)/human epidermal growth factor receptor 2 (HER2)(−) subtype of breast cancer.

## 2 Materials and methods

### 2.1 Materials

Actinomycin D, trichostatin A, cycloheximide, blasticidin, and puromycin were purchased from FUJIFILM Wako Pure Chemical Corporation (Osaka, Japan). Doxycycline and β‐nicotinamide mononucleotide (NMN) were purchased from Merck KGaA (Darmstadt, Germany) and Tokyo Chemical Industry Co., Ltd. (Tokyo, Japan), respectively.

### 2.2 Cell culture

All cell lines were obtained from the American Type Culture Collection (Manassas, VA, USA) or the Japanese Collection of Research Bioresources (JCRB, Osaka, Japan). The cells were cultured in their respective media (Table S1). All cell lines were authenticated using short tandem repeat analysis within 3 years. Authentications were confirmed by a 100% match compared with the reference short tandem repeat profiles from the JCRB cell bank. Mycoplasma tests were performed using MycoAlert Mycoplasma Detection Kit (Lonza, Basel, Switzerland) to exclude mycoplasma contamination.

### 2.3 RNA interference

shRNA was expressed using the CS‐RfA‐ErTBsd (TetOFF responsive) or CS‐RfA‐EP (constitutive) lentiviral vector [21]. Target sequences for nontargeting control and each gene were obtained from Mission shRNA library (Merck KGaA) (Table S2). siRNA (FlexiTube siRNA) was purchased from Qiagen (Venlo, the Netherlands). The transfection of siRNA (50 nm) was performed using Lipofectamine RNAiMAX Transfection Reagent (Thermo Fisher Scientific Inc., Waltham, MA, USA), according to the manufacturer's instructions.

### 2.4 Western blotting

Western blotting was performed as described [22]. The primary antibodies utilized in the study are listed in Table S3. Western blot data were quantified using ImageJ software (version 1.53 k, National Institutes of Health, Bethesda, MD, USA, https://imagej.net/ij/) to compare band densities.

### 2.5 Cell proliferation and viability assay

The cells were seeded at a density of 5 × 10^4^ or 1 × 10^5^ cells in six wells. Total cell number was counted with Countess II Automated Cell Counter (Thermo Fisher Scientific Inc.) on the indicated day. Cell viability was assessed by trypan blue staining.

### 2.6 Cell cycle analysis

Cells were fixed with 70% ethanol and stained with 10 μg·mL^−1^ propidium iodide (Merck KGaA) and 10 μg·mL^−1^ ribonuclease A (Merck KGaA) for 30 min at 37 °C. Images of stained nuclei were captured from at least 10 fields per well using CQ1 Confocal Quantitative Image Cytometer (Yokogawa Electric Corporation, Tokyo, Japan) and analyzed for DNA content distribution using Cell Pathfinder software (Yokogawa Electric Corporation) after discarding debris. Then, mean per well was quantified.

### 2.7 Quantification of metabolites

Analytical methods for the measurement of cationic and anionic metabolites were employed using capillary electrophoresis coupled with time‐of‐flight mass spectrometry. For metabolome analysis, the migration time of each peak was corrected using internal standards and authentic materials for migration time correction. Putative metabolites were identified based on the *m/z* and corrected migration times acquired from a standard mixture analyzed under the mas capillary electrophoresis conditions. The measurement data were processed with peak processing software (G2201AA Agilent Chemstation software for CE and Analyst QS software for TOF‐MS ver1.1). The peak area of each metabolite was normalized to that of the appropriate internal standard. The values of relative area were then further normalized by the sample weight.

### 2.8 Gene expression analysis

RNA extraction and quantitative reverse transcription PCR (qRT‐PCR) were performed as described [23], with minor modifications. The cDNA samples were mixed with specific primers (Table S4) and PowerUp SYBR Green Master Mix (Thermo Fisher Scientific Inc.) and amplified using QuantStudio 3 (Thermo Fisher Scientific Inc.), according to the manufacturer's instructions. Ct values of target genes were normalized to the TATA‐binding protein gene, and relative mRNA expression to control was shown.

### 2.9 Reporter assay

The reporter assay was performed as described previously [24]. Briefly, firefly luciferase reporter, WWP and WWP Pst [24], as well as the internal control of the *Renilla* luciferase reporter plasmid (pGL4.75 hRluc/CMV), were introduced into the cells using Lipofectamine 2000 Transfection Reagent (Thermo Fisher Scientific Inc.). At 48 h after transfection, luciferase activities were determined using Dual Luciferase Assay Kit (Promega). Firefly luciferase activity was normalized to *Renilla* luciferase activity.

### 2.10 Blue native PAGE and in‐gel CI activity assay

The procedures were performed according to a protocol [25]. Digitonin (1.7 mg·mL^−1^)‐treated whole cell lysates were extracted with n‐dodecyl‐β‐maltoside (protein:detergent ratio (g) = 1:2] in aminocaproic acid buffer [1.5 m aminocaproic acid and 50 mm Bis‐Tris (pH 7)] supplemented with protease inhibitors for 10 min on ice and centrifuged at 200 000 ***g*** for 30 min at 4 °C. Cleared lysates (5 μg protein) were mixed with 1/10 volume of native loading buffer [750 mm aminocaproic acid, 50 mm Bis‐Tris (pH 7), 0.5 mm EDTA, and 5% G‐250 Coomassie brilliant blue] and separated on a 4%–16% native PAGE.

The gel was incubated with CI substrate buffer [5 mm Tris/HCl (pH 7.4), 0.15 mm NADH, and 3 mm nitroblue tetrazolium] for 20 min at room temperature. The reaction was stopped, and the gel was fixed with 50% methanol and 10% acetic acid. Densitometric analysis was performed by measuring the band intensities of the activity based on the captured images using imagej software. After subtracting the background, the intensities were normalized with Coomassie G250 staining of the amount of protein loaded.

### 2.11 NAD ^+^/NADH measurement

Intracellular NAD^+^ and NADH were determined using NAD^+^/NADH Assay Kit‐WST (Dojindo Laboratories, Kumamoto, Japan), according to the manufacturer's instructions. Briefly, 5.0 × 10^5^ cells were lysed with NAD^+^/NADH extraction buffer and filtered using an ultracentrifugal filter. To calculate the amount of NADH, 50% of the filtrate was incubated at 60 °C for 1 h to decompose NAD^+^. All filtrates were enzymatically treated at 37 °C for 1 h to produce water‐soluble formazan. Sample absorbance was measured at 450 nm using Varioskan LUX (Thermo Fisher Scientific Inc.). The amount of NAD^+^ was determined by subtracting the amount of NADH assayed from the total amount of NAD^+^/NADH.

### 2.12 Determination of mitochondrial membrane potential and ATP levels

Mitochondrial membrane potential (ΔΨm) and intracellular ATP levels were measured using the Mito‐ID^®^ Membrane Potential Cytotoxicity Kit (Enzo Life Sciences, Farmingdale, NY, USA) and ATP determination kit (Thermo Fisher Scientific), respectively, as described previously [17].

### 2.13 Detection of intracellular ROS levels

The procedure for measuring intracellular ROS levels using 2′, 7′‐dichlorodihydrofluorescein diacetate (H_2_DCF; Thermo Fisher Scientific) was described previously [17].

To measure mitochondrial superoxide production, cells were incubated with 4 μm MitoSOX Red (Thermo Fisher Scientific) for 20 min at 37 °C in the dark. After washing, cells were collected from the culture plate with TrypLE Express (Thermo Fisher Scientific) and suspended in phosphate‐buffered saline containing 1% bovine serum albumin (Merck KGaA) and 0.5 μg·mL^−1^ 4′,6‐diamidino‐2‐phenylindole (DAPI, FUJIFILM Wako Pure Chemical Corporation). Following washing, cells were immediately analyzed using MoFlo Astrios EQ (Beckman Coulter) for MitoSOX Red (excitation 405 nm, emission 546/20 nm) and DAPI (excitation 405 nm, emission 448/59 nm). Excitation at 405 nm was specifically utilized for the selective detection of mitochondrial superoxide. After gating by cell size and granularity on the FSC/SSC plot, the mean fluorescence intensity of MitoSOX Red from 10 000 discrete cells unlabeled with DAPI was calculated using Kaluza Analysis 2.1 (Beckman Coulter). The level of mitochondrial superoxide was presented as the relative mean fluorescent intensity compared to the control level. Antimycin A (Merck KGaA) and 10‐(6′‐ubiquinolyl)decyltriphenylphosphonium bromide [17] were used to modulate ROS production in mitochondria.

To analyze the HyPer (Cyto and Mito) response to intracellular H_2_O_2_, cells were infected with the HyPer (Cyto or Mito) retrovirus [26], and stably infected cells were obtained by neomycin (Merck KGaA) selection. The cells were seeded at a density of 1.0 × 10^4^ cells into a 96‐well optical flat bottom plate. Following treatment or siRNA transfection, HyPer fluorescence was monitored using a CQ1 confocal quantitative image cytometer (Yokogawa Electric Corporation) with excitation at 405 or 488 nm and emission at 525 nm. Images were captured from at least 10 fields per well and analyzed using Cell Pathfinder software (Yokogawa Electric Corporation). The HyPer response was evaluated as the fluorescence ratio (ex 488/ex 405) after subtracting background, and the mean per well was quantified.

### 2.14 Measurement of oxygen consumption rate

Oxygen consumption rate (OCR) was measured using the Extracellular OCR Plate Assay Kit (Dojindo) according to the manufacturer's instructions. Briefly, 5 × 10^3^ cells were plated in a single well of a 96‐well black plate. 48 h after siRNA transfection, the cells were treated with an oxygen probe at 37 °C for 30 min in the culture medium. Subsequently, mineral oil was added dropwise. The fluorescence intensity was recorded (at excitation and emission wavelengths of 500 and 650 nm, respectively) at 10‐min intervals using the Varioskan Lux (Thermo Fisher Scientific) microplate reader. The OCR was calculated by analyzing the kinetic profiles obtained from the measurements.

### 2.15 Measurement of lactate secretion

The lactate levels were measured using the Lactate Assay Kit‐WST (Dojindo) according to the manufacturer's instructions. Briefly, 7.2 × 10^4^ cells were plated in a single well of a 12‐well plate. 48 h after siRNA transfection, the cells were treated with 22.5 mm 2‐deoxy‐d‐glucose or 1.25 μm oligomycin, or both in culture medium at 37 °C for 5 h. After incubation, the culture medium was diluted 10 times with water and used for analysis. The respective working solutions were incubated with the supernatants for 30 min at 37 °C, and then, the absorbance was measured at 450 nm using the Varioskan Lux (Thermo Fisher Scientific) microplate reader.

### 2.16 Immunocytochemistry

The immunocytochemistry procedure was essentially the same as that previously reported [27]. The antibodies against Flag and UQCRFS1 listed in Table S3 were used for this assay.

### 2.17 Expression vectors and viral transfection‐based cell establishment

The cDNAs for human *SIRT3*, *SIRT6*, *SIRT7*, and *SOD2* were amplified from the cDNA of human mammary epithelial cells and inserted into the CSII‐CMV‐MCS‐IRES2‐Bsd lentiviral vector (CSII vector) [26] or pMXs‐IP retroviral vector [28] with Flag tag sequences. A catalytically inactive SIRT7 mutant (SIRT7 H187Y) was generated using PrimeSTAR Mutagenesis Basal Kit (Takara Bio Inc., Shiga, Japan). Flag‐tagged NADH oxidase from *Lactobacillus brevis* (*Lb*NOX) and mitochondrial‐targeted *Lb*NOX (mito*Lb*NOX) were amplified from pUC57‐*Lb*NOX (Addgene plasmid # 75285) and pUC57‐mito*Lb*NOX (Addgene plasmid # 74448) [29], gifts from Dr. Vamsi Mootha, and cloned into the CSII‐TetOne vector (TetON responsive). The CSII‐TetOne vector was engineered by replacing the internal ribosome entry site and blasticidin resistance gene of CSII vector with TetOne cassette and SV40 promoter‐driven puromycin resistance gene from pRetroX‐TetOne‐Puro vector (Takara Bio Inc.). All constructs were confirmed by DNA sequencing.

Virus production and infection were performed as described [24]. Infected cells were selected, cultured in a medium supplemented with 10 μg·mL^−1^ blasticidin or 1 μg·mL^−1^ puromycin, and used for experiments.

### 2.18 Measurement of nascent protein synthesis

Cells were first cultured in DMEM medium without methionine for 1 h and then with a methionine analog, l‐azidohomoalanine (AHA, 50 μm), for 24 h to incorporate AHA into nascent proteins. The cells were lysed in RIPA buffer [10 mm Tris/HCl (pH 7.4), 150 mm NaCl, and 0.5% NP‐40] supplemented with protease inhibitor cocktail (Merck KGaA), sonicated, and centrifuged. Protein concentration was determined in the supernatant. Then, 200 μg of lysate was crosslinked to alkyne‐derivatized biotin (Thermo Fisher Scientific Inc.) by copper (I)‐catalyzed azide‐alkyne cycloaddition using Click‐iT Protein Reaction Buffer Kit (Thermo Fisher Scientific Inc.), according to the manufacturer's instructions. AHA‐labeled and biotin‐crosslinked proteins were isolated by affinity pulldown with FG streptavidin beads (Tamagawa Seiki, Nagano, Japan). Matrix‐bound proteins were washed with wash buffer [10 mm HEPES–NaOH (pH 7.9), 50 mm KCl, 1 mm EDTA, and 10% glycerol] and eluted with SDS sample buffer [2% SDS, 100 mm dithiothreitol, 60 mm Tris (pH6.8), 10% glycerol, and 0.1% bromophenol blue]. Affinity‐purified fractions were separated by SDS/PAGE and immunoblotted with antibodies.

### 2.19 CUT&RUN analysis

CUT&RUN was performed using the CUT&RUN Assay Kit (Cell Signaling Technology) following the manufacturer's instructions. Briefly, cells were harvested, washed, bound to activated concanavalin A‐coated magnetic beads, and then permeabilized. The bead–cell complex was incubated overnight with either anti‐acetyl‐histone H3 (Lys18) antibody (Cell Signaling Technology) or normal rabbit immunoglobulin (Agilent) at 4 °C. Subsequently, cells were washed with digitonin buffer, resuspended in digitonin buffer containing pAG‐MNase, and incubated for 1 h at 4 °C. After releasing DNA fragments into STOP buffer, DNA fragments were purified using NucleoSpin® Gel and PCR Clean‐up (Takara Bio) according to the manufacturer's instructions. Acetylation of H3K18 on the *p21* ^*Cip1*^ genomic locus was quantified by qPCR using the primers listed in Table S4.

### 2.20 Chromatin immunoprecipitation assay

Chromatin immunoprecipitation (ChIP) was performed as described [28], with slight modifications. Chromatin was sonicated using BIORUPTOR‐One (BM Equipment Co., Ltd., Tokyo, Japan) to generate 200–600 bp DNA fragments. Anti‐acetylated lysine 18 of histone H3 (H3K18Ac; Cell Signaling Technology, Inc., Danvers, MA, USA) was used for ChIP. Normal rabbit immunoglobulin (Agilent, Santa Clara, CA, USA) served as control. Percentage input was calculated by normalizing the Ct values between ChIP‐quantitative PCR (qPCR) and qPCR using input samples and expressed as fold‐change relative to control.

### 2.21 Clinical database analysis

The Kaplan–Meier method was used to perform survival analysis on the Molecular Taxonomy of Breast Cancer International Consortium (METABRIC) dataset, accession number EGAS00001001753, deposited at European Genome‐Phenome Archive. Based on the *z*‐score of mRNA expression relative to all samples (log microarray), as well as the status of estrogen and progesterone receptors and HER2, the samples were grouped and subjected to log‐rank test analysis using cBioPortal for Cancer Genomics.

### 2.22 Statistical analysis

All results were reported as the mean ± standard deviation from ≥3 independent samples with *P < 0.05 and **P < 0.01, unless otherwise noted. Comparisons between two groups were analyzed using the two‐tailed Student's *t*‐test. For multiple comparisons, data were analyzed using one‐way ANOVA with *post hoc* Dunnett's or Bonferroni's test to identify datasets that differed from the control data. A *P*‐value <0.05 was considered statistically significant.

## 3 Results

### 3.1 CI function mediated by NDUFV1 plays an important role in sustaining cell cycle progression in cancer cells

**Fig. 1:**
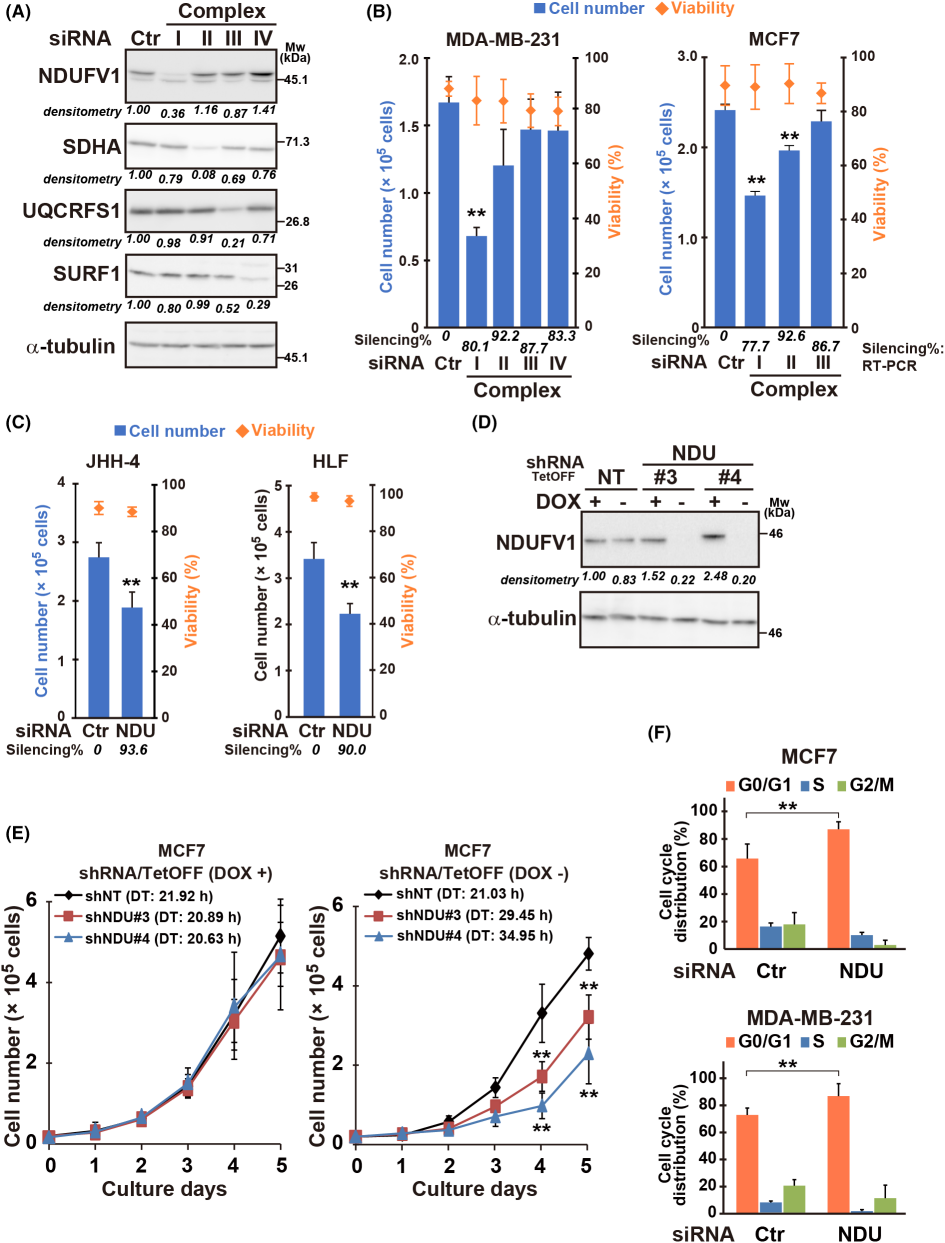
Complex I function mediated by NDUFV1 is important for cell cycle progression in cancer cells. (A) Western blotting with the indicated antibodies in MDA‐MB‐231 cells after 48 h of treatment with siRNAs for the major catalytic subunits of the electron transport chain complexes (I, *NDUFV1*; II, *SDHA*; III, *UQCRFS1*; IV, *SURF1*). Ctr, negative control siRNA. α‐Tubulin is the loading control. Silencing efficiency of each siRNA was evaluated based on the band intensity quantified by imagej software, which was normalized to that of α‐tubulin and shown relative to Ctr. Representative results from three independent experiments are shown. (B, C) Cell proliferation and viability (B, MDA‐MB‐231 and MCF7; C, JHH‐4 and HLF) after transfection with the indicated siRNAs for 72 h: NDU, *NDUFV1*. Silencing efficiencies of siRNAs are indicated in Table S5. (D, E) Doxycycline (DOX)‐responsive (TetOFF) shRNA [NDU (NDFUV1) or NT (nontarget control) (Table S2)]‐expressing cells were obtained from MCF7 cells by lentiviral transduction of the constructs (Materials and methods). Cells were incubated for 48 h in the presence (+) or absence (−) of DOX (1.0 μg·mL^−1^) and *NDUFV1* knockdown was validated by western blotting. Representative blots from three independent experiments are shown (D). Growth curve and doubling time. 2 × 10^4^ cells were seeded in the presence or absence of DOX; the number of viable cells was counted on each culture day. Doubling time (DT) was calculated from the slope of the graph (E). (F) Cell cycle distribution of siRNA‐transfected MCF7 and MDA‐MB‐231 cells. Bars and plots represent the means ± standard deviation from three independent experiments with measurements in triplicate in each experiment. For statistical analysis, two‐tailed Student's *t*‐test (C and F) and one‐way ANOVA Dunnett's multiple comparisons test among group (B and E) were used; **P < 0.01.

ETC activity was reported to regulate cancer cell proliferation [12, 13, 14, 15, 16]; however, the mechanisms involved and cancer type specificity are unclear. In this study, we focused on breast and liver cancer and determined which of the four ETC complexes was critical for cell proliferation. The major catalytic subunits of each complex: I; (NADH:ubiquinone oxidoreductase core subunit V1 [*NDUFV1*]), II; *SDHA*, III; *UQCRFS1*, and IV; *SURF1*, were knocked down in cells using siRNAs (Table S5). The silencing of *NDUFV1* significantly decreased cancer cell number without affecting viability (Fig. 1A–C; Fig. S1A). Thus, the importance of CI function, catalyzed by *NDUFV1*, was suggested in cell proliferation control.

Although cell proliferation was affected by the knockdown of other complexes to some extent, *NDUFV1* knockdown impaired cell proliferation in all four cell lines tested—two each of breast cancer (MDA‐MB‐231 and MCF7) and hepatocellular carcinoma (HLF and JHH‐4). *NDUFV1* knockdown with two independent shRNAs suppressed cell proliferation (Fig. 1D,E). Cell cycle analysis indicated that the cell cycle was arrested at the G_1_/S phase (Fig. 1F; Fig. S1B). Cell proliferation arrest due to CI inhibition could be due to limited aspartate synthesis [14, 18]. However, *NDUFV1* knockdown did not lower cellular aspartate (Table S6). Supplementation of aspartate and/or pyruvate in the culture medium was unable to rescue cell cycle arrest in the two hepatocyte cell lines, which expressed SLC1A3, an aspartate and glutamate transporter (Fig. S1C–E). These findings suggest that cell cycle arrest was caused by reasons other than limited amount of aspartate.

### 3.2 *p21* ^*Cip1*^ expression is upregulated when *NDUFV1* is silenced in cancer cells

**Fig. 2:**
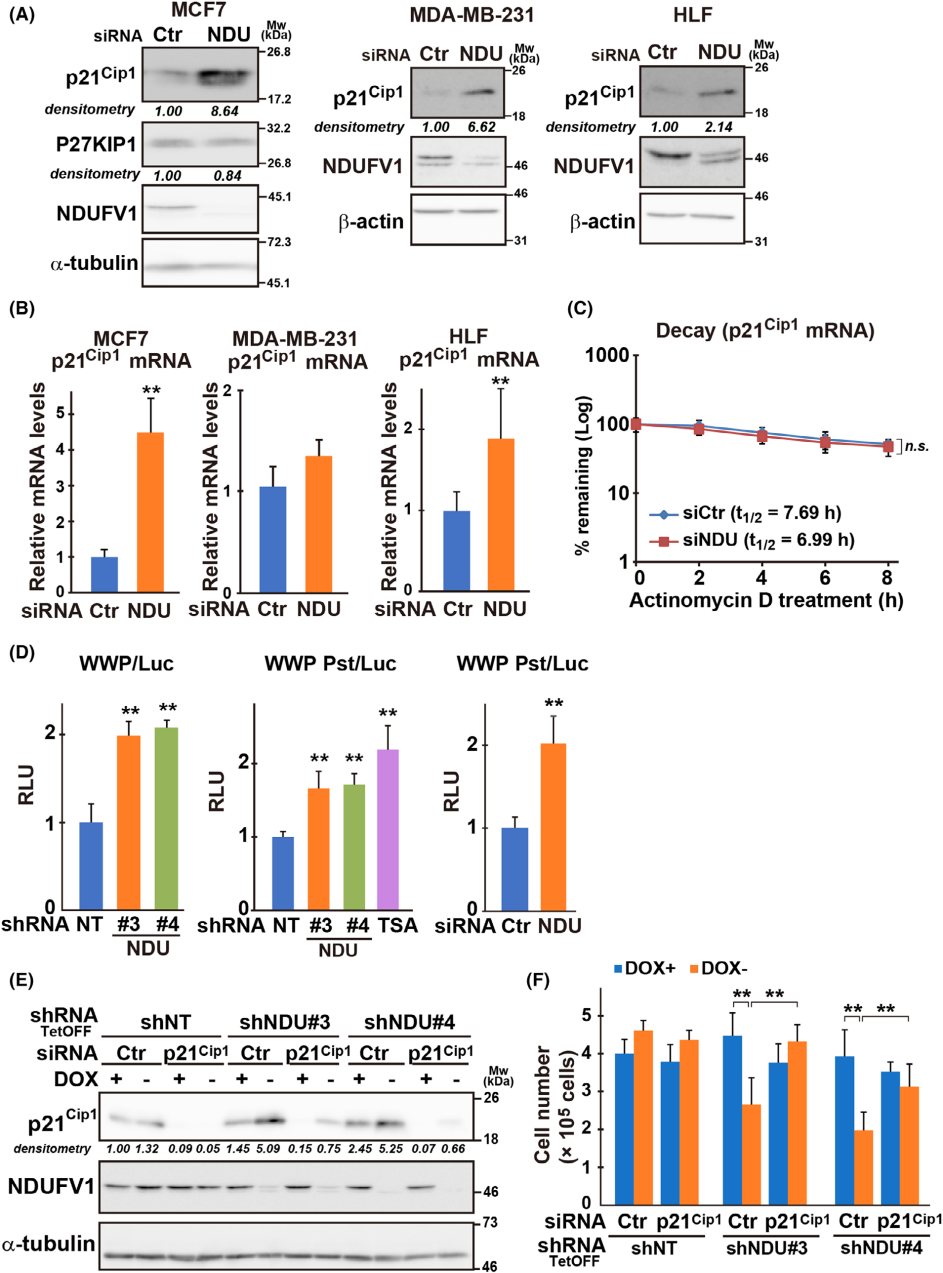
*NDUFV1* downregulation upregulates *p21* ^*Cip1*^ expression in cancer cells. (A–C) Cells were incubated with siRNAs for 48 h: NDU, siRNA for *NDUFV1*; Ctr, negative control siRNA. Western blotting with the indicated antibodies. Band intensities measured with ImageJ are shown as relative to the control (Ctr). Representative blots from three independent experiments are shown. α‐Tubulin and β‐actin are the loading controls (A). The mRNA levels of *p21* ^*Cip1*^ examined using quantitative reverse transcription PCR (qRT‐PCR). Values are relative to control (Ctr) (B). Cells were treated at 0 h with actinomycin D (1 μg·mL^−1^) and at each time point, the amount of *p21* ^*Cip1*^ mRNA was determined by qRT‐PCR. The percentages of remaining *p21* ^*Cip1*^ mRNA were graphed (value at 0 h was defined as 100%). The half‐life (*t*_1/2_) of mRNA was calculated from the slope of the graph (C). (D) Reporter activity assessed in *NDUFV1*‐silenced MCF7 cells with shRNA or siRNA as in A and Fig. 1D. Cells were transfected and incubated with WWP reporter plasmids (Materials and methods) together with the internal control for 48 h. A ratio to the control (NT, Ctr) is shown. Trichostatin A (TSA, 0.5 μm) was used as positive control. RLU, relative luciferase units. (E, F) DOX‐responsive (TetOFF) shRNA‐expressing MCF7 cells as in Fig. 1D were transfected with siRNA for *p21* ^*Cip1*^ or negative control siRNA (Ctr) in the presence (+) or absence (−) of DOX (1 μg·mL^−1^). After 48 h, knockdown effects were validated as in A. Band intensities are shown as relative to control (shNT/Ctr, DOX+). Representative blots from three independent experiments are shown. α‐Tubulin is the loading control (E). Cell proliferation and viability were assessed after 72 h of transfection (F). Bars and plots represent the means ± standard deviation from three independent experiments with measurements in triplicate in each experiment. Two‐tailed Student's *t*‐test (B and C) and one‐way ANOVA following by Dunnett's multiple comparisons test among group (D and F); **P < 0.01, n.s., not significance.

Recently, we found that mitochondrial dysfunction induces cyclin‐dependent kinase inhibitor *p21* ^*Cip1*^ and *P27KIP1* expression [27]. In this study, *NDUFV1* downregulation upregulated *p21* ^*Cip1*^, whereas *P27KIP1* levels were unchanged (Fig. 2A; MCF7). *p21* ^*Cip1*^ upregulation was observed in 6 and 9/16 tumorigenic and nontumorigenic breast and hepatocyte cell lines at mRNA and protein levels, respectively, indicating that this response is conserved in several cell lines (Table S1, Figs S2, S3). The difference between the upregulation positive and negative cell lines is unclear at this stage. It is unlikely that the upregulation was associated with glucose and pyruvate availability in the culture medium (Table S1). The upregulation appeared at mRNA and protein levels, or at either, depending on the cell line. For example, in MCF7 and HLF cells, *NDUFV1* knockdown increased *p21* ^*Cip1*^ mRNA and protein levels (Fig. 2A,B; MCF7, HLF). The increase in mRNA was likely due to transcriptional regulation because mRNA stability was virtually unaffected by *NDUFV1* knockdown (Fig. 2C). Reporter genes incorporated downstream of the upstream regions of the *p21* ^*Cip1*^ gene showed *NDUFV1* knockdown activated transcription from the proximal promoter (Fig. 2D). The activation levels were comparable to those mediated by a typical transcriptional activator of *p21* ^*Cip1*^, trichostatin A (TSA). In contrast to MCF7, in MDA‐MB‐231 cells, the increase in *p21* ^*Cip1*^ protein levels was not accompanied by increase in mRNA levels (Fig. 2A,B; MDA‐MB‐231), suggesting that *p21* ^*Cip1*^ was post‐transcriptionally upregulated. Therefore, *NDUFV1* downregulation potentially upregulates *p21* ^*Cip1*^ expression transcriptionally and post‐transcriptionally. In further studies, we mainly used MCF7 cells, in which *p21* ^*Cip1*^ was upregulated at the mRNA and protein levels.

Notably, the cell proliferation defects mediated by *NDUFV1* downregulation did not occur in the presence of *p21* ^*Cip1*^ siRNA (Fig. 2E,F; Fig. S4). Thus, upregulated *p21* ^*Cip1*^ contributed significantly to cell cycle arrest upon *NDUFV1* downregulation. These results suggest that CI activity suppresses *p21* ^*Cip1*^ expression, thereby supporting cell cycle progression and contributing to tumorigenesis.

### 3.3 Decrease in NAD ^+^/NADH signals to *p21* ^*Cip1*^ upregulation under the downregulation of CI activity

**Fig. 3:**
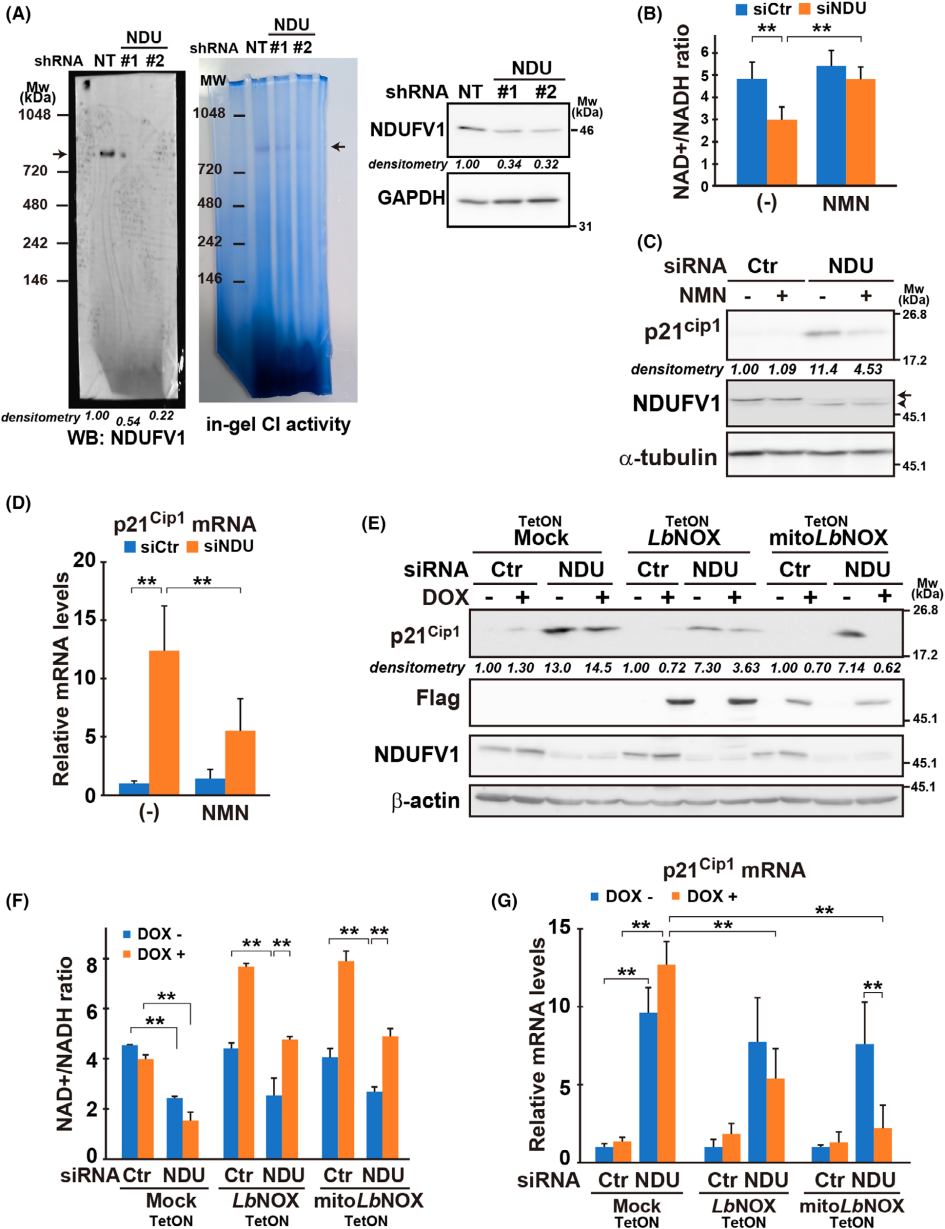
Decrease in the NAD^+^/NADH ratio signals to upregulate *p21* ^*Cip1*^ expression when Complex I activity is silenced. (A) MCF7 cells constitutively expressing shNDUFV1 (NDU) #1 or #2 and nontarget control shRNA (NT) were established. Immediately after selection and confirmation of *NDUFV1* knockdown by western blotting (GAPDH: the loading control), the cells were treated and extracted with detergents and subjected to Blue native PAGE followed by western blotting with anti‐NDUFV1 antibody (WB: NDUFV1) or in‐gel Complex I (CI) activity assay (Materials and methods). Representative results from two independent experiments are shown. (B–D) MCF7 cells were treated with siRNA (Ctr, control; NDU, *NDUFV1*) in the presence or absence of 1 mm β‐nicotinamide mononucleotide (NMN), and the NAD^+^/NADH ratio was evaluated after 24 h (B). The levels of protein (C) and mRNA (D) were examined after 48 h by western blotting and qRT‐PCR, respectively. Values are relative to control (Ctr/−). Representative blots from three independent experiments are shown. Arrow and arrowhead indicate the position of NDUFV1 and unknown contingent bands, respectively. α‐Tubulin is the loading control. (E–G) MCF7 cells were infected with DOX‐responsive lentivirus constructs (TetON) expressing Flag‐tagged native (*Lb*NOX), mitochondrial‐targeted *Lb*NOX (mito*Lb*NOX), or control (Mock). After 48 h of transfection with siRNA with (+) or without (−) DOX (0.5 μg·mL^−1^), western blotting (E) and qRT‐PCR (G) were performed. Representative blots from three independent experiments are shown. β‐Actin is the loading control. Values are relative to control (Ctr/DOX−). The NAD^+^/NADH ratio was determined after treatment with siRNA for 24 h in the presence or absence of DOX (F). Bars represent the means ± standard deviation from three independent experiments with measurements in triplicate in each experiment. One‐way ANOVA with Bonferroni *post hoc* test among group; **P < 0.01.

CI, consisting of 45 subunits, catalyzes NADH oxidation––the first step of the ETC reaction. Among the subunits, the 51‐kDa subunit of NDUFV1 provides NADH‐binding and flavin mononucleotide (FMN)‐ and Fe–S‐biding sites, which are critical for CI activity, and transfers electrons from NADH to ubiquinone, thereby regenerating NAD^+^ and maintaining the NAD^+^/NADH ratio. The in‐gel CI activity assay revealed that NADH dehydrogenase activity was reduced to 56% (#1) and 52% (#2) of the control (NT) following *NDUFV1* knockdown with the shRNAs in a high molecular weight complex containing NDUFV1 (Fig. 3A; arrows). Therefore, downregulation of *NDUFV1* primarily causes shortage of NAD^+^ or imbalance of the NAD^+^/NADH ratio, which possibly results in *p21* ^*Cip1*^ upregulation. Alternatively, ROS overproduction or ATP deficiency could mediate the phenomenon indirectly.

To identify a mediator for *p21* ^*Cip1*^ upregulation under *NDUFV1* downregulation, we analyzed typical ETC‐related parameters, such as the NAD^+^/NADH ratio, ROS and ATP levels, and mitochondrial membrane potential (ΔΨm). Measurement of these parameters revealed that all values, except the NAD^+^/NADH ratio (whole‐cell ratio), were stable under knockdown of ETC components, including *NDUFV1* (Fig. 3B; (−) and Fig. S5). An uncoupler CCCP or complex III inhibitor antimycin A (AMA) varied the parameters correspondingly to their effects. We measured ROS levels with three widely used probes: H_2_DCF detected ROS broadly, MitoSOX Red detected O_2_^−^, and HyPer (Cyto)/(Mito) detected cytosolic/mitochondrial H_2_O_2_. All probes yielded similar results indicating that ROS levels were within normal range when ETC components were knocked down; however, AMA, which interrupts ETC instead of knockdown of a component, remarkably elevated the levels (Fig. S5D; MitoSOX Red/AMA). Thus, it was unlikely that ROS‐mediated or oxidative stress‐related signals mediated *p21* ^*Cip1*^ upregulation and caused cell proliferation arrest under *NDUFV1* downregulation.

We also evaluated the impact of *NDUFV1* knockdown on cellular metabolism. Under *NDUFV1*‐downregulated conditions, cellular oxygen consumption rate decreased significantly (Fig. S6A). However, total ATP levels were sustained (Figs S5B, S6B; Ctr vs NDU), suggesting that cells compensated the deficiency in respiratory activity for energy production by activating an alternative pathway, mostly glycolysis, considering that ATP levels were minimized in the presence of 2‐deoxy‐d‐glucose (2‐DG) and oligomycin together. In particular, oligomycin treatment revealed that the cancer cells stimulate glycolytic ATP production excessively upon inhibition of respiration—*NDUFV1* downregulation stimulated glycolysis synergistically with oligomycin (Fig. S6B). Consistently, lactate secretion increased under these conditions (Fig. S6C). Upon inhibition of the glycolytic pathway in the presence of 2‐DG, complex II‐driven mitochondrial ATP production may be activated in *NDUFV1*‐silenced cells. Regarding TCA cycle intermediates, the levels of α‐ketoglutarate, citrate, and succinate in *NDUFV1*‐silenced cells were comparable to those in the control (Table S6). Fumarate, with the lowest levels, was under detection limit in the cells. Overall, the TCA cycle was not affected significantly in *NDUFV1*‐silenced cells.

Together, these findings suggested that the *NDUFV1* downregulation impacts on the NAD^+^/NADH, but not ROS and ATP levels, accompanying adaptation of cellular metabolism to CI inhibition. Therefore, we hypothesized that when *NDUFV1* was knocked down, a decrease in the NAD^+^/NADH ratio triggered the *p21* ^*Cip1*^ upregulation. To verify this hypothesis, we supplied NMN, a precursor of NAD^+^, to the medium. In the presence of NMN, the NAD^+^/NADH ratio was maintained under *NDUFV1* knockdown conditions (Fig. 3B; Fig. S5A; NMN). In addition, *p21* ^*Cip1*^ mRNA and protein upregulation were remarkably attenuated (Fig. 3C,D; Fig. S7A), supporting the hypothesis that *p21* ^*Cip1*^ is upregulated in response to a decrease in the NAD^+^/NADH ratio. Next, we introduced NADH oxidase from *Lactobacillus brevis* (*Lb*NOX) into cells to manipulate the NAD^+^/NADH ratio [29]. We used two types of *Lb*NOX: (1) the native form that is localized throughout the cell (*Lb*NOX) and (2) the engineered form that is localized to the mitochondria with a mitochondrial targeting sequence (mito*Lb*NOX) (Fig. S8). Both forms of *Lb*NOX were equally effective in increasing the whole‐cell ratio of NAD^+^/NADH (Fig. 3F; Fig. S7B). Notably, *Lb*NOX, in particular, the mitochondrial form, markedly inhibited the upregulation of *p21* ^*Cip1*^, (Fig. 3E,G; Fig. S7C). The effects of NMN and *Lb*NOX on *p21* ^*Cip1*^ induction provide strong evidence that a decrease in NAD^+^ or the NAD^+^/NADH ratio is critical for upregulating *p21* ^*Cip1*^. Thus, NAD^+^ levels are key determinants of *p21* ^*Cip1*^ expression.

### 3.4 Translational regulation of *p21* ^*Cip1*^ levels by SIRT3 at the downstream of CI activity

NAD^+^ not only acts as a redox coenzyme in metabolic reactions but also serves as a substrate for several enzymes. Our results indicate that the role of NAD^+^ as a substrate for NAD^+^‐dependent deacetylase family, sirtuin (SIRT), is important for regulating *p21* ^*Cip1*^ expression. When seven mammalian SIRTs were silenced individually with siRNAs (Table S5), each member potentially contributed to the regulation of *p21* ^*Cip1*^ expression at mRNA or protein level, depending on the cell line (Fig. S9A). In particular, the knockdown of *SIRT3* and *SIRT7* upregulated the protein levels of *p21* ^*Cip1*^ in all three cell lines studied. Therefore, we focused on the roles of SIRT3 and SIRT7 in the upregulation of *p21* ^*Cip1*^ under *NDUFV1*‐silenced conditions. Interestingly, SIRT3 and SIRT7 appeared to regulate *p21* ^*Cip1*^ expression in mechanistically different ways.

**Fig. 4:**
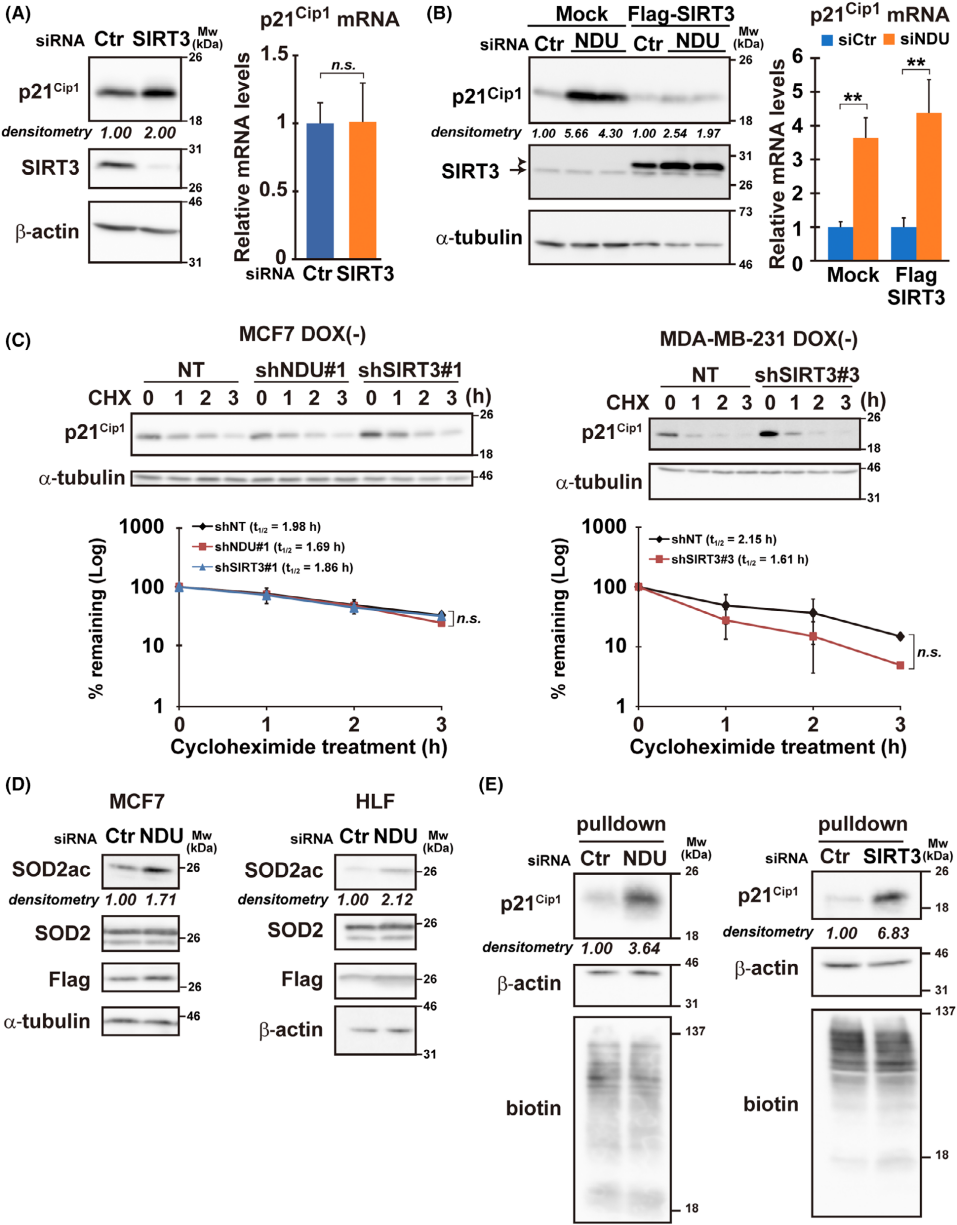
Translation of *p21* ^*Cip1*^ is promoted by the downregulation of SIRT3 activity when Complex I activity is silenced. (A) Western blotting and qRT‐PCR with MCF7 cells transfected with siRNA for *SIRT3* (silencing efficiency is indicated in Table S5) or negative control siRNA (Ctr) for 48 h. Values are relative to control (Ctr). β‐Actin is the loading control. (B) Western blotting and qRT‐PCR with MCF7 cells expressing Flag‐tagged SIRT3 (Flag‐SIRT3) or control (Mock) after 48 h of transfection with *NDUFV1* siRNA (NDU) or negative control siRNA (Ctr). Values are relative to control (Ctr). Arrowhead and arrow indicate exogenous and endogenous SIRT3, respectively. α‐Tubulin is the loading control. (C) MCF7 and MDA‐MB‐231 cells expressing DOX‐responsive (TetOFF) shRNA (Table S2) were cultured for 72 h in the absence of DOX. After treatment with cycloheximide (100 μg·mL^−1^) for the indicated time, p21^Cip1^ was quantified by western blotting. After normalization to α‐tubulin values, the remaining amounts were plotted (value at 0 h was defined as 100%). The half‐life (*t*_1/2_) of protein was calculated from the slope of the graph. α‐Tubulin is the loading control. (D) The acetylation levels of SOD2 (SOD2ac) were examined using western blotting in cells expressing Flag‐tagged SOD2 after 48 h of transfection with siRNA. The band intensity of SOD2ac was normalized to that of SOD2 and shown as relative to control (Ctr). α‐Tubulin and β‐actin are the loading controls. (E) MCF7 cells were transfected with the indicated siRNAs, and nascent protein synthesis was examined after 24 h (Materials and methods). The affinity‐purified fractions were examined using western blotting with the indicated antibodies. The band intensity of p21^Cip1^ was normalized to that of β‐actin and shown as relative to control (Ctr). Representative results from three independent experiments are shown. β‐Actin is the loading control. In western blotting, representative blots from three independent experiments are shown. Bars and plots represent the means ± standard deviation from three independent experiments with measurements in triplicate in each experiment. Two‐tailed Student's *t*‐test (A and C) and one‐way ANOVA following by Bonferroni multiple comparisons test among group (B); **P < 0.01, n.s., not significance.

*SIRT3* knockdown increased *p21* ^*Cip1*^ protein levels (Fig. 4A; Fig. S9A), and *SIRT3* overexpression inhibited the *NDUFV1* knockdown‐mediated increase in *p21* ^*Cip1*^ protein levels (Fig. 4B; Fig. S9B) and not mRNA levels (Fig. 4A,B). Because the cycloheximide chase assay revealed that *p21* ^*Cip1*^ protein was not stabilized by knocking down *SIRT3* (Fig. 4C; Fig. S9C), SIRT3 most likely regulated *p21* ^*Cip1*^ translation, thereby downregulating its level. Judging from the increase in the acetylation of SOD2, a validated substrate of SIRT3 deacetylation, the deacetylase activity of SIRT3 was suggested to be downregulated under *NDUFV1*‐silenced conditions (Fig. 4D).

Collectively, SIRT3 deacetylase activity decreases when *NDUFV1* is knocked down, leading to the translational upregulation of *p21* ^*Cip1*^. To prove this hypothesis, we assayed the translational efficiency of *p21* ^*Cip1*^ under *SIRT3*‐silenced and control conditions. Nascent cellular proteins were first labeled with a methionine surrogate, to which a tag was chemically conjugated in the following step. Then, labeled and tagged proteins were pulled down using the tag. Compared to the control, the *SIRT3* knockdown, as well as the *NDUFV1* knockdown, remarkably accelerated *p21* ^*Cip1*^ translation (Fig. 4E). Studies have reported that *p21* ^*Cip1*^ translation was regulated by a eukaryotic translation initiation factor EIF2A and EIF4E‐binding protein 4E‐BP [30, 31]. Given that the phosphorylation status or function of both proteins was likely modified under *NDUFV1* and *SIRT3* knockdown conditions (Fig. S9D), these proteins are potential downstream targets of the NAD^+^–SIRT3 pathway in the translational regulation of *p21* ^*Cip1*^.

### 3.5 Transcriptional regulation of *p21* ^*Cip1*^ expression by SIRT7 at the downstream of CI activity

**Fig. 5:**
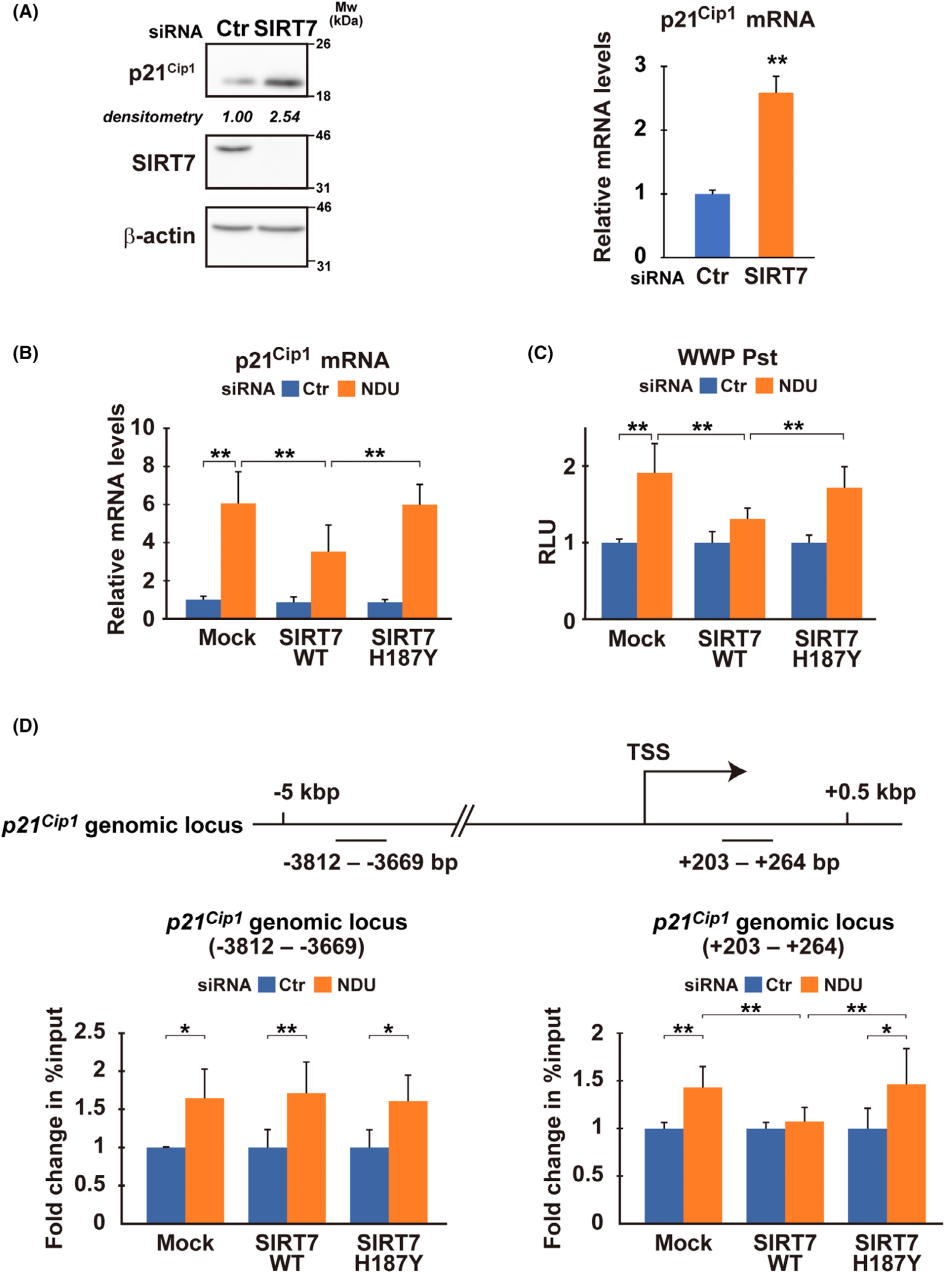
Transcription of *p21* ^*Cip1*^ is suppressed by SIRT7 activity under Complex I activity. (A) Western blotting and qRT‐PCR performed in MCF7 cells after 48 h of transfection with siRNA for *SIRT7* or negative control siRNA (Ctr). Values are relative to control (Ctr). Representative blots from three independent experiments are shown. β‐Actin is the loading control. (B–D) MCF7 cells expressing Flag‐tagged SIRT7 (wild‐type, SIRT7 WT; a catalytically inactive mutant, SIRT7 H187Y), or control (Mock) were treated with *NDUFV1* (NDU) siRNA or negative control siRNA (Ctr). After 48 h, the mRNA levels of *p21* ^*Cip1*^ were examined by qRT‐PCR (B). Values are relative to control (Mock/Ctr). In (C), the WWP Pst reporter was transfected into cells with the internal control and siRNA, and after 48 h, reporter activities were evaluated as in Fig. 2D. Fold changes normalized to the control (Ctr) are graphed. In (D), ChIP was performed to examine the enrichment of acetyl H3K18 on the *p21* ^*Cip1*^ locus after treatment with siRNA for 30 h. Precipitated DNA was analyzed by qPCR using primers specific to the *p21* ^*Cip1*^ locus (Table S4), as indicated in the map. Fold changes are normalized to control (Ctr). TSS, transcription start site. Bars represent the means ± standard deviation from three independent experiments with measurements in triplicate in each experiment. Two‐tailed Student's *t*‐test (A) and one‐way ANOVA with Bonferroni's multiple comparisons test among group (B–D); *P < 0.05, **P < 0.01.

Knockdown experiments suggested that SIRT7 also regulates *p21* ^*Cip1*^ expression (Fig. S9A). In contrast to *SIRT3*, *SIRT7* knockdown affected *p21* ^*Cip1*^ levels both at protein and mRNA levels (Fig. 5A; Fig. S9A). Thus, it is likely that SIRT7 regulates *p21* ^*Cip1*^ expression at the transcriptional level. We substantiated this possibility in an experiment overexpressing *SIRT7* (Fig. S10A), which inhibited the *NDUFV1* knockdown‐mediated increase in *p21* ^*Cip1*^ promoter activity, as well as mRNA expression (Fig. 5B,C; Fig. S11A; SIRT7 WT). The inhibitory effect was not observed with SIRT6, another nuclear‐localized sirtuin (Fig. S10B). Importantly, unlike the wild‐type, a catalytically inactive SIRT7 mutant (SIRT7 H187Y) was ineffective in inhibiting *p21* ^*Cip1*^ upregulation (Fig. 5B,C). Thus, *NDUFV1* knockdown likely downregulated SIRT7 activity due to the decrease in the NAD^+^/NADH ratio, leading to *p21* ^*Cip1*^ transcriptional upregulation.

Given its almost exclusive localization in the chromatin‐enriched nuclear fraction and role as an NAD^+^‐dependent deacetylase specific to acetylated lysine 18 of histone 3 (H3K18Ac) [32], SIRT7 deacetylases H3K18Ac around the *p21* ^*Cip1*^ gene, thereby negatively affecting transcription. To test this hypothesis, we examined H3K18Ac around the *p21* ^*Cip1*^ promoter. CUT&RUN analysis preliminarily located a potential target of SIRT7 at the locus (from +203 to +264 bp) (Fig. S10C). We performed the ChIP assay focusing on the locus and found that *NDUFV1* downregulation increased H3K18Ac, which was reversed by overexpressing wild‐type SIRT7 (Fig. 5D; Fig. S11B; from +203 to +264 bp). The catalytically inactive H187Y mutant was ineffective. At the far upstream region (from −3812 to −3669 bp), *SIRT7* knockdown and overexpression had little impact on H3K18Ac levels (Fig. 5D; Figs S10C, S11B; from −3812 to −3669 bp), whereas *NDUFV1* downregulation increased H3K18Ac levels (Fig. 5D; Mock/Ctr vs. NDU). Thus, it is possible that although H3K18 is marked with acetylation broadly around the gene under *NDUFV1*‐silenced conditions, SIRT7 is specifically responsible for H3K18Ac from +203 to +264. Altogether, CI function of regenerating NAD^+^ supports SIRT7 deacetylase activity, which is necessary for keeping H3K18 deacetylated at the locus proximal to the promoter and suppressing *p21* ^*Cip1*^ transcription.

### 3.6 Expression levels of the main core subunits of CI correlate with the prognosis of patients with HR(+)/HER2(−) breast cancer

**Fig. 6:**
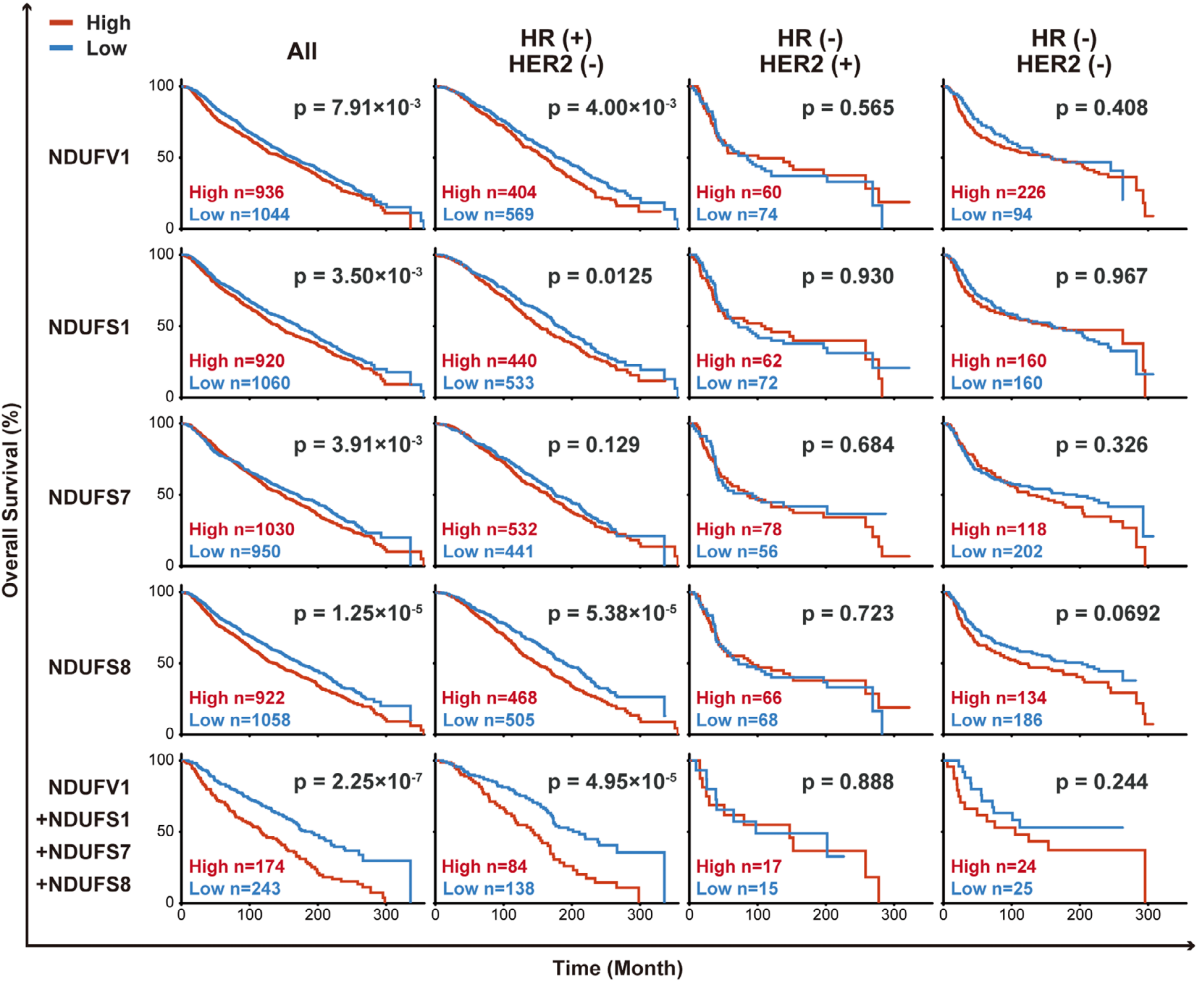
Expression levels of the core subunits of Complex I correlate with overall survival in patients with HR(+)/HER2(−) breast cancer. Kaplan–Meier plots with patients categorized by expression levels [H, high (*z*‐score >0), and L, low (*z*‐score ≤0)] of core subunits of electron transport chain complex I were obtained in each subtype of breast cancer using the METABRIC dataset through cBioPortal (described in Materials and methods); *n*, number of samples. The *P*‐value for the log‐rank test is indicated in each plot.

We examined whether CI functionality is associated with human cancer progression. We analyzed the correlation of CI expression levels with overall survival in patients with breast cancer. We focused on the core subunits of CI, which are sufficient for the assembly of the functional enzyme [33, 34], and covered seven nuclear‐encoded subunits [33, 35], the datasets of which are available at METABRIC. Kaplan–Meier plots indicated that high expression of 4/7 subunits, including *NDUFV1*, correlated with poor prognosis in patients, specifically, with estrogen/progesterone HR(+)/HER2(−) subtype of breast cancer (Fig. 6). The simultaneous high expression of these four units predicted an aggravated outcome.

**Fig. 7:**
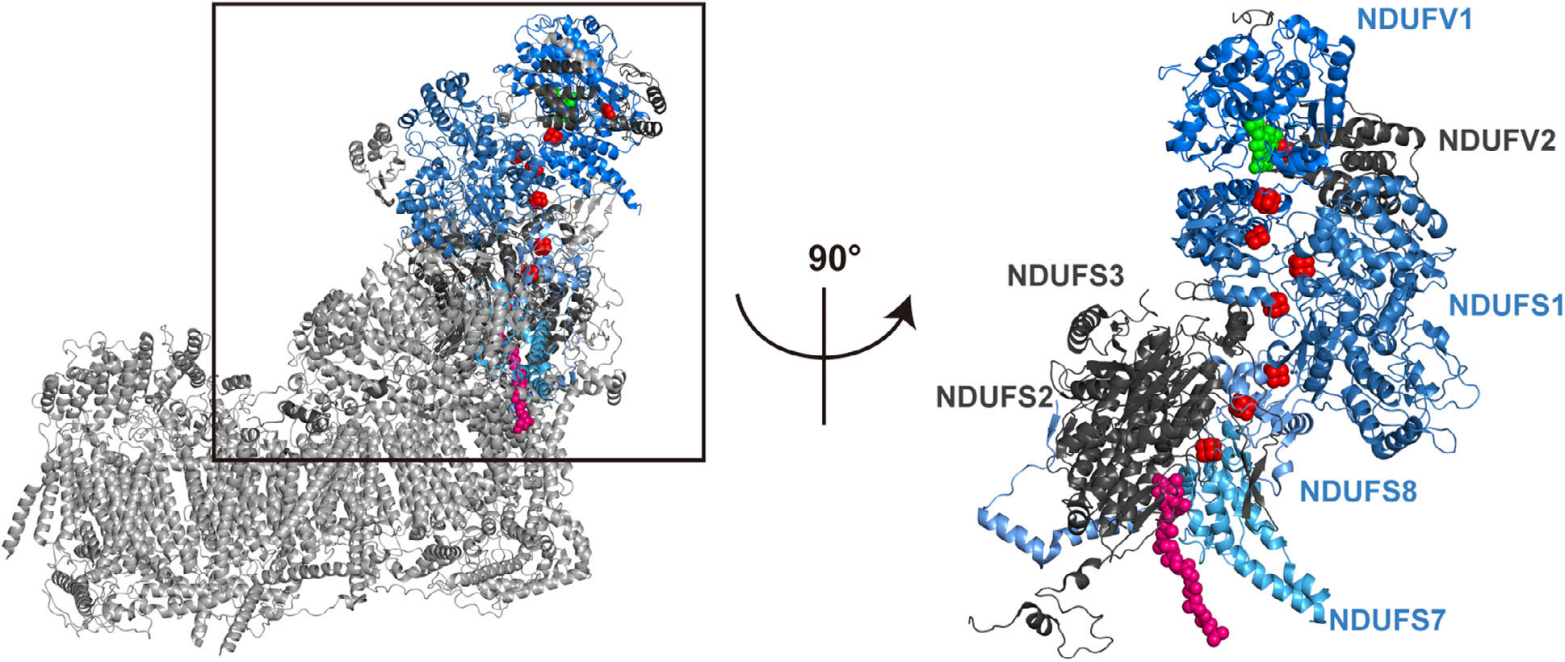
Cryoelectron microscopy structure of electron transport chain Complex I. Bovine electron transport chain complex I (CI) with active ubiquinone (Protein Data Bank ID: 7QSK) [36] was superposed onto the human CI (Protein Data Bank ID: 5XTD) [37], after which bovine CI was removed (left). The subunits, comprising the electron transfer path, which contains flavin mononucleotide (FMN, green), eight Fe–S clusters (red), and ubiquinone (magenta), are boxed, rotated 90°, and shown on the right. The four subunits, expression levels of which correlated with prognosis in patients with breast cancer (Fig. 6), are shown in blue, and others are shown in gray. The structures are visualized using PyMOL (Version 2.5.7, The PyMOL Molecular Graphics System, Schrödinger LLC).

Notably, all four subunits (NDUFV1, NDUFS1, NDUFS7, and NDUFS8) contain 1–3 Fe–S clusters––cofactors essential for electron transport [34]; NDUFV1 also contains NADH and FMN binding sites. However, *NDUFV2*, *NDUFS2*, and *NDUFS3*, whose expression levels have little impact on patient survival of any subtype of breast cancer (Fig. S12A), contain no Fe–S clusters or play no role in the main path of electron transport. CI contains eight Fe–S clusters that are arranged such that their configuration defines the path of electron transfer, which begins at the NADH‐binding site of NDUFV1 and terminates at the ubiquinone binding site of NDUFS2 and NDUFS7 [34] (Fig. 7). Among the eight clusters, seven are contained in the four subunits, whereas one is contained in the NDUFV2 subunit, which constitutes an off‐pathway that bifurcates from the main path and barely contributes to ETC [38]. Thus, these analyses uncovered the possible association of the CI functionality—the NAD^+^‐regenerating capability accompanied by electron transfer by NDUFS1, NDUFS7, and NDUFS8—with cancer progression in patients with HR(+)/HER2(−) breast cancer. From a clinical point of view, the expression levels of the core CI subunits, which constitute the main flow of electron path, have potential prognostic value for patients with the breast cancer subtype. Similarly, CI functionality potentially contributed to the progression of melanoma and head and neck tumors (Fig. S12B).

## 4 Discussion

We found that *NDUFV1* downregulation of CI causes cell cycle arrest in subsets of liver and breast cancer cell lines. We targeted the core subunit and decreased oxidoreductase activity from CI using RNAi. Thus, we could interfere with the NAD^+^/NADH ratio and associate NADH dehydrogenase activity directly with cell proliferation control. Our results suggest that the NADH dehydrogenase activity of regenerating NAD^+^ maintains SIRT activity and suppresses antiproliferative *p21* ^*Cip1*^ expression in cancer cells. When NAD^+^‐regenerating activity was downregulated, in parallel with the decrease in the NAD^+^/NADH ratio, the activities of SIRTs decreased, resulting in the upregulation of *p21* ^*Cip1*^ and cell cycle arrest. In summary, NAD^+^‐SIRT‐p21^Cip1^ emerged as one of the pathways that switch cell cycle progression in response to NAD^+^‐regenerating activity, thereby integrating cellular metabolism with cell proliferation control.

The role of CI in cell proliferation control has been addressed in several studies, where CI was disrupted by chemical inhibitors and not gene knockdown [14, 15, 18]. Unlike RNAi‐mediated *NDUFV1* downregulation, CI inhibition by chemical inhibitors, such as rotenone, interrupted electron flow and enhanced ROS production from CI [39]. Under such CI‐inhibited conditions, the cell cycle was arrested in cancer cells, such as leukemia [18], osteosarcoma, glioblastoma, and lung carcinoma [14], due to a deficiency in aspartate. Because aspartate is a key biosynthetic precursor of proteins, purine nucleotides, and pyrimidine nucleotides, the cells cannot proliferate. A key finding of these studies is that NAD^+^ is most limiting for the *de novo* synthesis of aspartate in cancer cells. These findings underline the importance of NAD^+^ regeneration by CI in aspartate biosynthesis. However, under our experimental conditions, exogenous supplementation of aspartate or pyruvate or measurement of aspartate argued against the possibility that NAD^+^ was limiting for aspartate synthesis.

Interestingly, a decrease in the NAD^+^/NADH ratio was reported to increase the ratios of ADP or AMP to ATP, whereby AMPK was activated and induced senescence in fibroblastic cells in a p53‐ or p16‐dependent manner [20]. We examined the possible involvement of NAD^+^–AMPK–p53 pathway in our study with MCF7 cells, in which p53 is wild‐type. However, AMPK was not activated when *NDUFV1* was knocked down (Fig. S13). Furthermore, the induction of *p21* ^*Cip1*^ was observed in MDA‐MB‐231 and HLF cells, in which p53 is mutated, suggesting that the induction was p53‐independent and unrelated to the NAD^+^–AMPK–p53 pathway. Our observations suggest roles for SIRT3 and SIRT7, both of which are NAD^+^‐dependent class III histone deacetylases implicated in various diseases, including cancer [40]. Interestingly, SIRT3, the main deacetylase in mitochondria, regulated *p21* ^*Cip1*^ expression at the translational level, whereas SIRT7, which is predominantly nuclear, regulated the transcription of *p21* ^*Cip1*^ through histone deacetylation at H3K18, thereby avoiding the increase in p21^Cip1^ levels in cancer cells.

These findings indicate the importance of CI‐mediated NAD^+^ regeneration in cancer cell proliferation and tumorigenesis. However, the contribution of CI function to human tumorigenesis is unclear. Although mitochondrial and nuclear DNA mutations in the genes encoding CI subunits are found in various cancers, including breast cancer [41], the impacts of these mutations are either pro or antitumorigenic. The outcome possibly depends on tumor type or the severity and type of dysfunction caused by individual mutations [42, 43]. Importantly, our analysis of patient survival with breast cancer suggested that CI functionality contributes to cancer progression of the HR(+)/HER2(−) subtype. Thus, CI activity was considered protumorigenic in this breast cancer subtype. Additionally, CI functionality was potentially protumorigenic in head and neck tumors, and melanoma, but not liver cancer (Fig. S12B). Therefore, contribution of CI functionality to tumorigenesis is different between *in vivo* and *in vitro* settings and among cancer types.

Cellular metabolism is essential for cell survival and proliferation, and therefore, an attractive target for cancer therapy. However, successful development of metabolism‐based medicine for cancer treatment has been limited [44]. Cell‐autonomous and cell‐nonautonomous complexities pose challenges for targeting cellular metabolism therapeutically [45, 46]. Among them, the most fundamental challenge is that cancer cell metabolism is not inherently different from that of normal cells. Unless a particular metabolism‐related enzyme is mutated, which promotes tumor growth, metabolism is reprogrammed in cancer cells as in normal proliferating cells. This means that cancer cell metabolism cannot be a bona fide specific target and poses a real challenge to harness therapeutically [45]. Interference with cellular metabolism, which is prosurvival, inevitably destroys both cancer and normal cells.

As an alternative to inactivating prosurvival pathways, our results raise the possibility to activate antiproliferative pathways in cancer cells. Cells originally respond to irrelevant circumstances, including metabolically nonpermissive conditions, and stop proliferation with the aid of tumor suppressor functions. Given that tumor suppressor activity would be static but not toxic to cells, the strategy is possibly less detrimental to normal cells and therapeutically preferable to targeting prosurvival pathways. Inhibiting CI or decreasing intracellular NAD^+^ could be such a strategy that exploits the antiproliferative function of p21^Cip1^. The strategy would benefit patients with HR(+)/HER2(−) breast cancer, which accounts for nearly 70% of breast cancers. Similarly, SIRT3 or SIRT7 could be potential targets in a similar context.

Several CI‐targeting compounds are under clinical trials as anticancer drugs, following promising results in preclinical models [43, 47]. Metformin was related to a lower risk of cancer in patients with type 2 diabetes and expected to be repurposed as an anticancer agent; however, later studies reached conflicting or disappointing conclusions [43, 48, 49, 50]. Our study encourages the development of compounds that inhibit NADH dehydrogenase for a personalized therapy against HR(+)/HER2(−) breast cancer. Although it is necessary to minimize possible adverse effects, which can be predicted by referring to the phenotypes of inherited mitochondrial disorders caused by mutations of CI‐encoding genes [51], cancer therapy targeting NAD^+^‐regenerating CI activity would be a promising approach, especially HR(+)/HER2(−) breast cancer, and contribute to better patient prognosis.

## 5 Conclusions

Among the activities of the ETC complexes, the NADH dehydrogenase activity of CI was critical for cell proliferation in breast and liver cancer cells. Our analyses suggested that the NAD^+^/NADH ratio, which is maintained by the NAD^+^‐regenerating activity of the dehydrogenase, is necessary for NAD^+^‐dependent deacetylase SIRT3 and SIRT7 to suppress *p21* ^*Cip1*^ expression at the translational and transcriptional levels, respectively. Thus, the NAD^+^–SIRT3/7–p21^Cip1^ pathway couples cellular metabolism and proliferation, supporting cancer cell proliferation. Consistently, Kaplan–Meier analysis indicated that CI functionality contributes to cancer progression, especially that of the HR(+)/HER2(−) breast cancer subtype.

## Conflict of interest

The authors declare no conflict of interest.

## Author contributions

MH and KM: Investigation, validation, formal analysis, and writing (original draft). HN and MU: Investigation, validation, and formal analysis. FI: Methodology, writing (review and editing). MS: Conceptualization, supervision, and project administration.

## Peer review

The peer review history for this article is available at https://www.webofscience.com/api/gateway/wos/peer‐review/10.1002/1878‐0261.13808.

## Supporting information


**Fig. S1.** Effects of *NDUFV1* knockdown and aspartate and pyruvate supplementation in the culture medium on cell proliferation. (A) Representative phase contrast images of the cells shown in Fig. 1B and E. Scale bar: 50 μm. (B) Representative histograms of the cell cycle analysis shown in Fig. 1F. The cell cycle distribution percentages are indicated. (C) The *SLC1A3* protein levels were examined by western blotting after transfection with siRNA against *NDUFV1* (NDU) or negative control siRNA (Ctr) for 48 or 72 h. β‐actin is the loading control. (D, E) Hc3716‐h*TERT* hepatocytes (D) and HLF (E) were transfected with the indicated siRNAs with or without 10 mm aspartate or 1 mm pyruvate. After 72 h, the number of viable cells was counted. *P < 0.05, **P < 0.01.

**Fig. S2.** Screening of *NDUFV1* knockdown to induce *p21* ^*Cip1*^ mRNA expression. The *p21* ^*Cip1*^ mRNA levels were examined using qRT‐PCR after transfection with siRNA (Ctr, control; NDU, *NDUFV1*) for 48 h or after incubating cells expressing DOX‐responsive (TetOFF) shRNA for 48 h with (+) or without (−) doxycycline (DOX, 1 μg·mL^−1^). *P < 0.05, **P < 0.01. The results of MCF7, MDA‐MB‐231, and HLF cells are shown in Fig. 2B.

**Fig. S3.** Screening of *NDUFV1* knockdown to induce p21^Cip1^ protein expression. The *p21* ^*Cip1*^ protein levels were examined by western blotting after transfection with siRNA (Ctr, control; NDU, *NDUFV1*) for 72 h or after incubating cells expressing DOX‐responsive (TetOFF) shRNA for 72 h with (+) or without (−) doxycycline (DOX, 1 μg·mL^−1^). β‐actin, GAPDH, and α‐tubulin are the loading controls. The results of MCF7, MDA‐MB‐231, and HLF cells are shown in Fig. 2A.

**Fig. S4.** *p21* ^*Cip1*^ induction mediates cell proliferation arrest under *NDUFV1* knockdown in MDA‐MB‐231 cells. (A) Western blotting with the indicated antibodies using MDA‐MB‐231 cells after treatment with siRNAs against *NDUFV1* (NDU), *p21* ^*Cip1*^, negative control siRNA (Ctr) or combinations as indicated for 72 h. β‐actin is the loading control. (B) Cell proliferation and viability were assessed after transfection with siRNAs for 72 h. **P < 0.01.

**Fig. S5.** Effect of *NDUFV1* and other ETC component knockdown on mitochondrial membrane potential, NAD^+^/NADH, ATP, and ROS levels. (A) MDA‐MB‐231 cells were treated with siRNA (Ctr, control; NDU, *NDUFV1*) in the presence or absence of 1 mm β‐nicotinamide mononucleotide (NMN). The NAD^+^/NADH ratio was evaluated after 24 h. (B, C) ATP levels (B) and ΔΨm (C) were examined in MDA‐MB‐231 cells using an ATP determination kit and the Mito‐ID Membrane Potential Cytotoxicity kit, respectively, after transfection with siRNAs (I, *NDUFV1*; II, *SDHA*; III, *UQCRFS1*; IV, *SURF1*) for 48 h. Values were presented relative to the Ctr. Carbonyl cyanide 3‐chlorophenylhydrazone (CCCP, FUJIFILM Wako Pure Chemical Corporation) was used as a control to induce mitochondrial depolarization. (D) Intracellular ROS levels were analyzed with 2′, 7′‐dichlorodihydrofluorescein diacetate (H_2_DCFDA) and MitoSOX Red using flow cytometry after transfection with the indicated siRNA for 48 h. The mean fluorescence intensity of H_2_DCFDA and MitoSOX Red, obtained from at least 10 000 cells, was presented as a ratio to the Ctr. Antimycin A (AMA, 10 μm) and mitochondria‐specific antioxidant, 10‐(6′‐ubiquinolyl) decyltriphenylphosphonium bromide (MQ, 0.5 μm) were used to modulate the ROS levels in the mitochondria. To measure the HyPer response, cells were infected with retroviral constructs expressing HyPer (Cyto or Mito, materials and methods). After transfection with the indicated siRNA for 48 h, images were captured using a CQ1 confocal quantitative image cytometer and analyzed using Cell Pathfinder software. After subtracting background, the HyPer response was evaluated as the fluorescence ratio (ex 488/ex 405). The values are presented relative to the Ctr. H_2_O_2_ treatment (50 μm, 10 min) was employed as a positive control. **P < 0.01.

**Fig. S6.** *NDUFV1* knockdown shifts energy metabolism to glycolysis. (A) The oxygen consumption rate (OCR) was examined in MCF‐7 cells using the Extracellular OCR Plate Assay Kit after transfection with siRNA (Ctr, control; NDU, *NDUFV1*) for 48 h. Values are relative to control (Ctr). (B, C) ATP and (B) lactate (C) levels were examined in MCF‐7 cells using an ATP determination kit and Lactate Assay Kit‐WST, respectively. MCF‐7 cells were transfected with siRNA (Ctr, control; NDU, *NDUFV1*) for 48 h and then treated with 22.5 mm 2‐deoxy‐d‐glucose (2‐DG) or 1.25 μm oligomycin (Oligo) or both at 37 °C for 5 h. The cell lysate and the culture supernatants were used for ATP (B) and lactate (C) assays, respectively. Values are relative to control (Ctr, no treatment).

**Fig. S7.** Decrease in the NAD^+^/NADH ratio signals to upregulate *p21* ^*Cip1*^ expression in MDA‐MB‐231 cells. (A) Western blotting with the indicated antibodies in MDA‐MB‐231 cells after transfection with siRNAs against *NDUFV1* (NDU) or negative control siRNA (Ctr) for 72 h with or without 1 mm β‐nicotinamide mononucleotide (NMN). β‐actin is the loading control. (B, C) MDA‐MB‐231 cells were infected with doxycycline (DOX)‐responsive lentivirus constructs (TetOFF) expressing Flag‐tagged native (*Lb*NOX), mitochondrial‐targeted *Lb*NOX (mito*Lb*NOX). The NAD^+^/NADH ratio was determined after siRNA treatment for 24 h in the presence (+) or absence (−) of DOX (2 ng·mL^−1^) (B). Western blotting was performed 72 h after siRNA transfection with (+) or without (−) DOX (C). β‐actin is the loading control. **P < 0.01.

**Fig. S8.** The subcellular localization of *Lb*NOX and mito*Lb*NOX. The subcellular localization of *Lb*NOX and mito*Lb*NOX was examined by immunocytochemistry using antibodies against Flag and UQCRFS1 after seeding *Lb*NOX‐ or mito*Lb*NOX‐expressing MCF7 cells for 24 h with DOX (0.5 μg·mL^−1^). The cell nuclei were labeled with DAPI. Scale bar: 20 μm.

**Fig. S9.** Effect of SIRTs knockdown on *p21* ^*Cip1*^ protein and mRNA levels, and translational machinery. (A) The *p21* ^*Cip1*^ protein and mRNA levels were examined by western blotting and qRT‐PCR, respectively, after transfection with the indicated siRNA for 48 or 72 h. Silencing efficiencies of siRNAs are indicated in Table S5. β‐actin is the loading control. The band intensity values and the mRNA levels are shown relative to the Ctr. **P < 0.01. (B) MDA‐MB‐231 cells expressing Flag‐tagged SIRT3 (Flag‐SIRT3) or control (mock) were treated with *NDUFV1* (NDU) siRNA or negative control siRNA (Ctr). After 72 h, the protein levels of *p21* ^*Cip1*^ were examined by western blotting. α‐Tubulin is the loading control. (C) MCF‐7 cells expressing doxycycline (DOX)‐responsive (TetOFF) SIRT3#1, SIRT3#3, or non‐target control (NT) shRNA were cultured for 72 h with (+) or without (−) DOX (1 μg·mL^−1^). Knockdown of *SIRT3* was examined by western blotting. β‐Actin is the loading control. (D) MCF‐7 cells were incubated with siRNAs against *NDUFV1* (NDU), *SIRT3*, or negative control (Ctr) for 48 h. Western blotting with the indicated antibodies. Band intensities measured with ImageJ are shown as relative to the control (Ctr). β‐Actin is the loading control. An arrow and an arrowhead indicate the phosphorylated forms of 4E‐BP1 and 4E‐BP2/3, respectively.

**Fig. S10.** Effect of SIRT7 and SIRT6 overexpression on *p21* ^*Cip1*^ expression. (A) MCF7 cells were infected with lentiviral constructs expressing Flag‐tagged SIRT7 WT, SIRT7 H187Y (Materials and methods), or control (Mock). After selection, exogenous SIRT7 expression was confirmed by western blotting with the indicated antibodies. GAPDH was the loading control. (B) Cells expressing Flag‐tagged SIRT7 WT or control (Mock) were infected with retroviral vectors (Mock or Flag‐tagged SIRT6). After selection, exogenous SIRT6 and SIRT7 expression was confirmed as above. GAPDH was the loading control. *p21* ^*Cip1*^ mRNA expression was examined by qRT‐PCR after transfection with the indicated siRNA for 48 h. Values are presented relative to the control (Mock/Ctr). (C) CUT&RUN was performed to examine the enrichment of acetylated H3K18 on the *p21* ^*Cip1*^ genomic locus after 24 h of siRNA transfection (materials and methods). The fold changes normalized to the Ctr were plotted. TSS; transcription start site. *P < 0.05, **P < 0.01.

**Fig. S11.** Transcription of *p21* ^*Cip1*^ is suppressed by SIRT7 under CI activity in HLF cells. (A, B) HLF cells expressing Flag‐tagged wild‐type SIRT7 (SIRT7 WT) or control (mock) were treated with *NDUFV1* (NDU) siRNA or negative control siRNA (Ctr). After 48 h, the mRNA levels of *p21* ^*Cip1*^ were examined by qRT‐PCR (A). Values are relative to control (mock/Ctr). In (B), ChIP was performed to examine the enrichment of acetyl H3K18 on the *p21* ^*Cip1*^ locus after siRNA treatment for 54 h. The precipitated DNA was analyzed by qPCR using primers specific to the *p21* ^*Cip1*^ locus (Table S4), as indicated in the map. The fold changes were normalized to the Ctr. TSS; transcription start site. **P < 0.01.

**Fig. S12.** The impact of expression levels of CI subunits on overall survival in patients with breast cancer and other cancers. (A) Kaplan–Meier plots of the overall survival of patients with each subtype of breast cancer, categorized by the expression levels [high, *z*‐score >0; low, *z*‐score ≤0] of the indicated subunits, were obtained as in Fig. 6. (B) Kaplan–Meier plots with patient categorized by expression levels [High, *z*‐score >0; Low, *z*‐score ≤ 0] of the indicated subunits were obtained in each cancer type using the TCGA PanCancer Atlas dataset through cBioPortal (described in Materials and methods). *n*, number of samples.

**Fig. S13.** NAD^+^‐SIRT‐p21^Cip1^ pathway is independent of AMPK. Doxycycline (DOX)‐responsive (TetOFF) shRNA [NT, non‐target control; NDU, *NDFUV1*]‐expressing MCF7 cells were incubated for 72 h in the presence (+) or absence (−) of DOX (1.0 μg·mL^−1^). Western blotting was performed with the indicated antibodies. The loading control is α‐tubulin. The band intensities measured with ImageJ are shown relative to the control (NT/DOX+) after normalization using loading control.

**Table S1.** Cell lines used in the study.

**Table S2.** List of targeting sequences for shRNA.

**Table S3.** List of antibodies.

**Table S4.** Primer sequences for qPCR, ChIP, and CUT&RUN.

**Table S5.** Silencing efficiency of siRNAs.

**Table S6.** Quantification of aspartate and TCA cycle intermediates in MCF7 cells.

## Data Availability

The data that support the findings of this study are available from the corresponding author upon reasonable request.
