# Supplementary figures and images for: Respiratory complex I‐mediated NAD + regeneration regulates cancer cell proliferation through the transcriptional and translational control of p21 Cip1 expression by SIRT3 and SIRT7
Source: Mol Oncol. 2025 Jan 28;19(6):1775–96. doi: 10.1002/1878-0261.13808 (PMC12161471; doi:10.1002/1878-0261.13808)

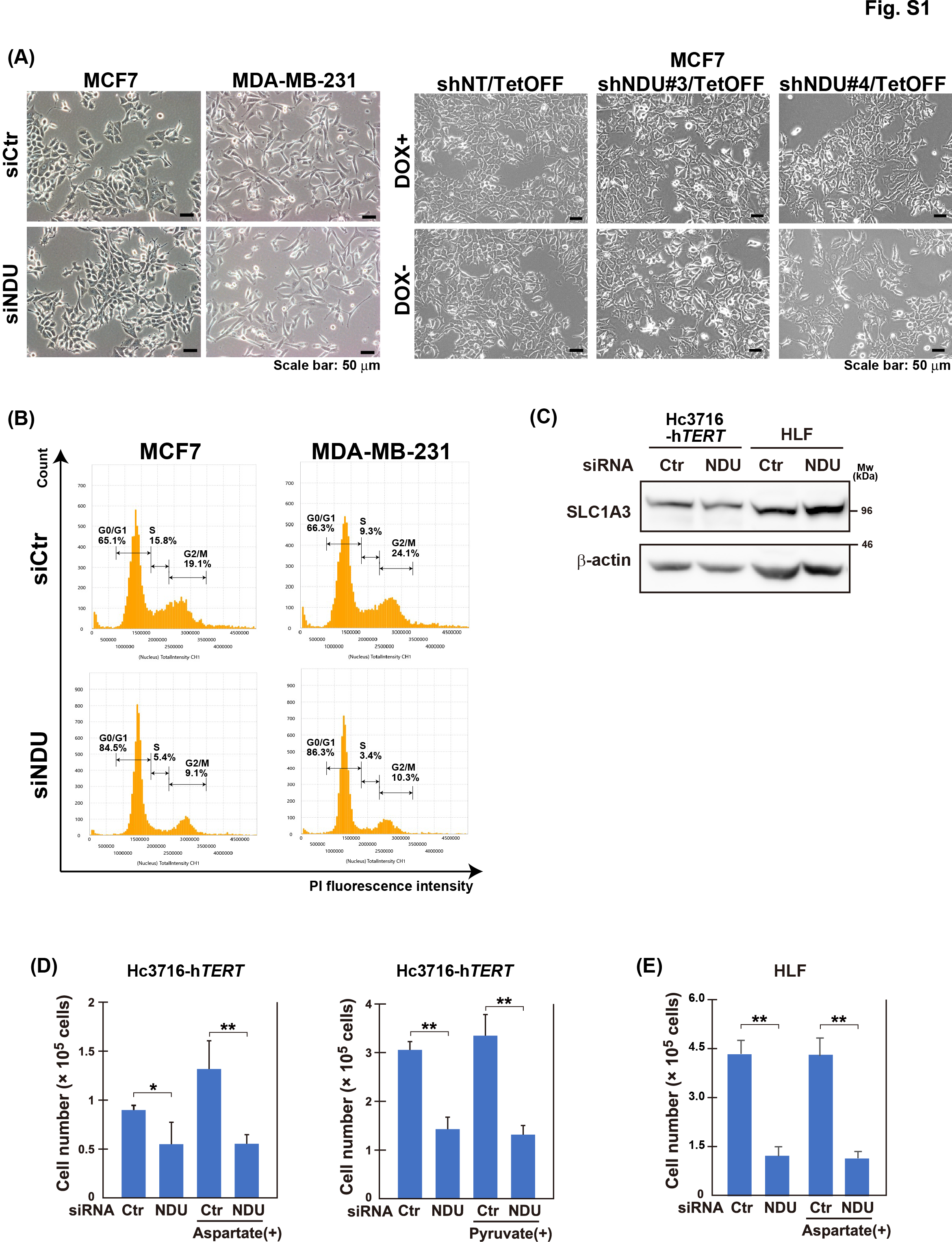

Supplement: Supplementary file 1 — Fig. S1. Effects of NDUFV1 knockdown and aspartate and pyruvate supplementation in the culture medium on cell proliferation. (A) Representative phase contrast images of the cells shown in Fig. 1B and E. Scale bar: 50 μm. (B) Representative histograms of the cell cycle analysis shown in Fig. 1F. The cell cycle distribution percentages are indicated. (C) The SLC1A3 protein levels were examined by western blotting after transfection with siRNA against NDUFV1 (NDU) or negative control siRNA (Ctr) for 48 or 72 h. β‐actin is the loading control. (D, E) Hc3716‐hTERT hepatocytes (D) and HLF (E) were transfected with the indicated siRNAs with or without 10 mm aspartate or 1 mm pyruvate. After 72 h, the number of viable cells was counted. *P < 0.05, **P < 0.01. [file MOL2-19-1775-s010.tif]

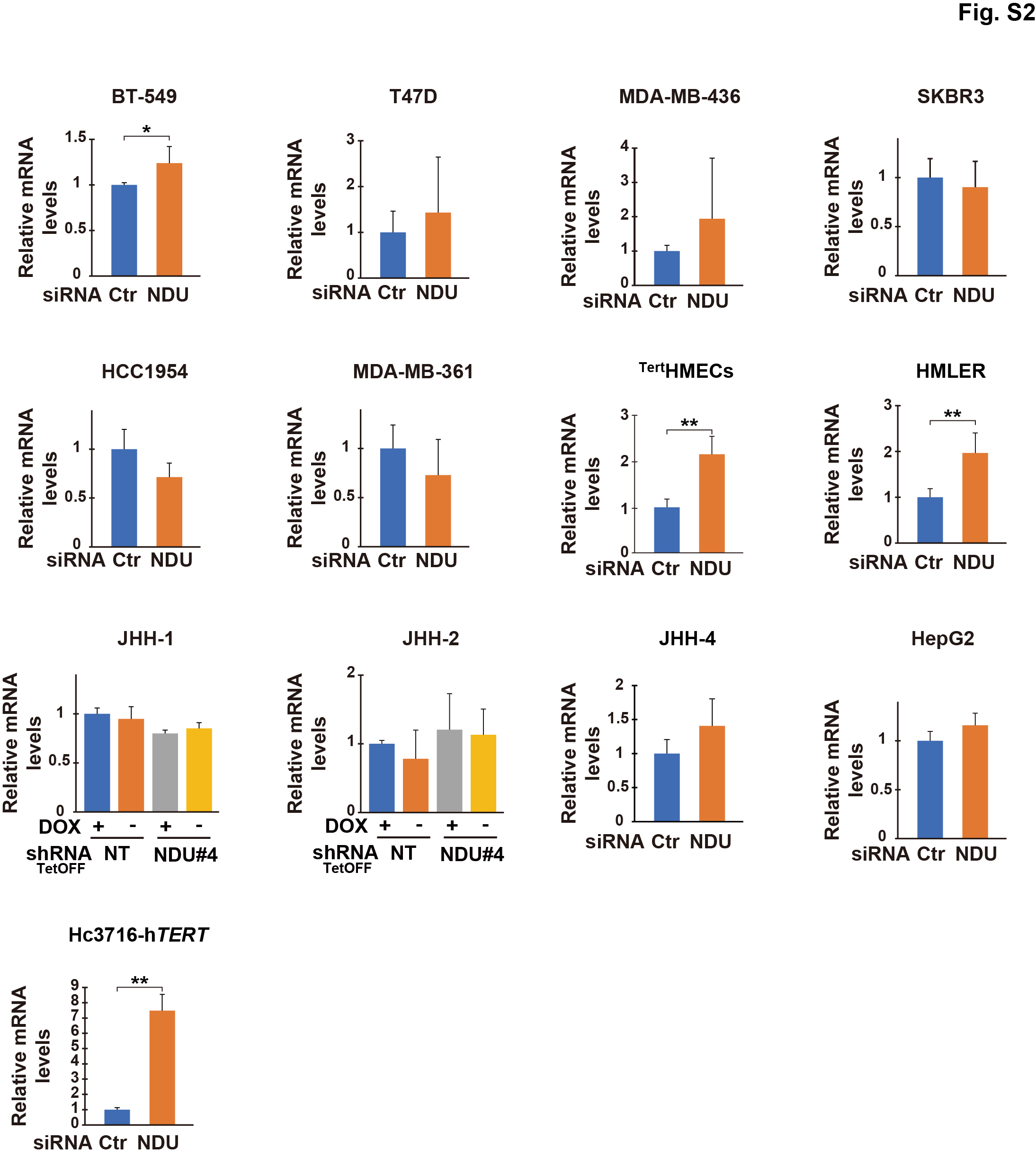

Supplement: Supplementary file 2 — Fig. S2. Screening of NDUFV1 knockdown to induce p21 Cip1 mRNA expression. The p21 Cip1 mRNA levels were examined using qRT‐PCR after transfection with siRNA (Ctr, control; NDU, NDUFV1) for 48 h or after incubating cells expressing DOX‐responsive (TetOFF) shRNA for 48 h with (+) or without (−) doxycycline (DOX, 1 μg·mL−1). *P < 0.05, **P < 0.01. The results of MCF7, MDA‐MB‐231, and HLF cells are shown in Fig. 2B. [file MOL2-19-1775-s011.tif]

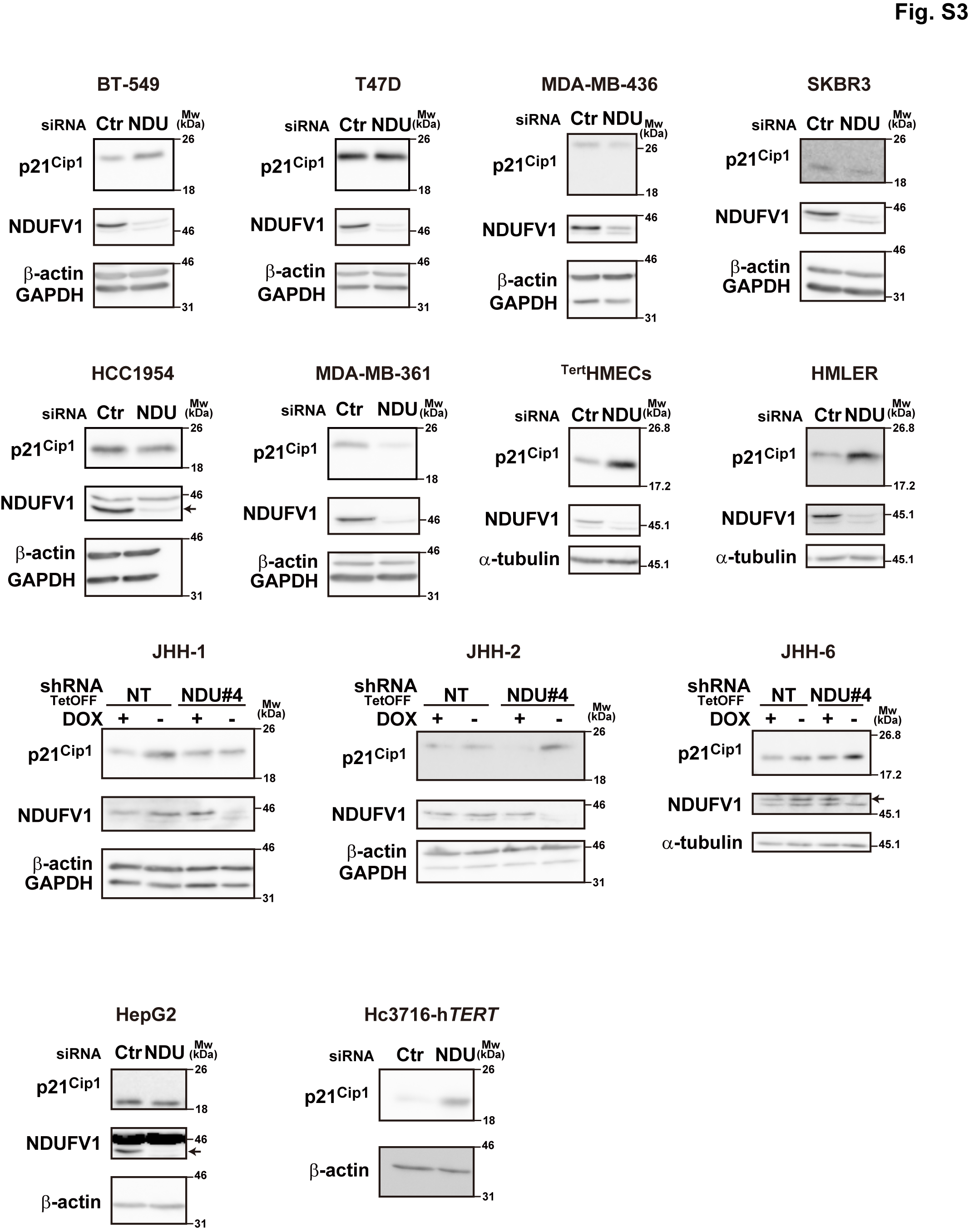

Supplement: Supplementary file 3 — Fig. S3. Screening of NDUFV1 knockdown to induce p21Cip1 protein expression. The p21 Cip1 protein levels were examined by western blotting after transfection with siRNA (Ctr, control; NDU, NDUFV1) for 72 h or after incubating cells expressing DOX‐responsive (TetOFF) shRNA for 72 h with (+) or without (−) doxycycline (DOX, 1 μg·mL−1). β‐actin, GAPDH, and α‐tubulin are the loading controls. The results of MCF7, MDA‐MB‐231, and HLF cells are shown in Fig. 2A. [file MOL2-19-1775-s006.tif]

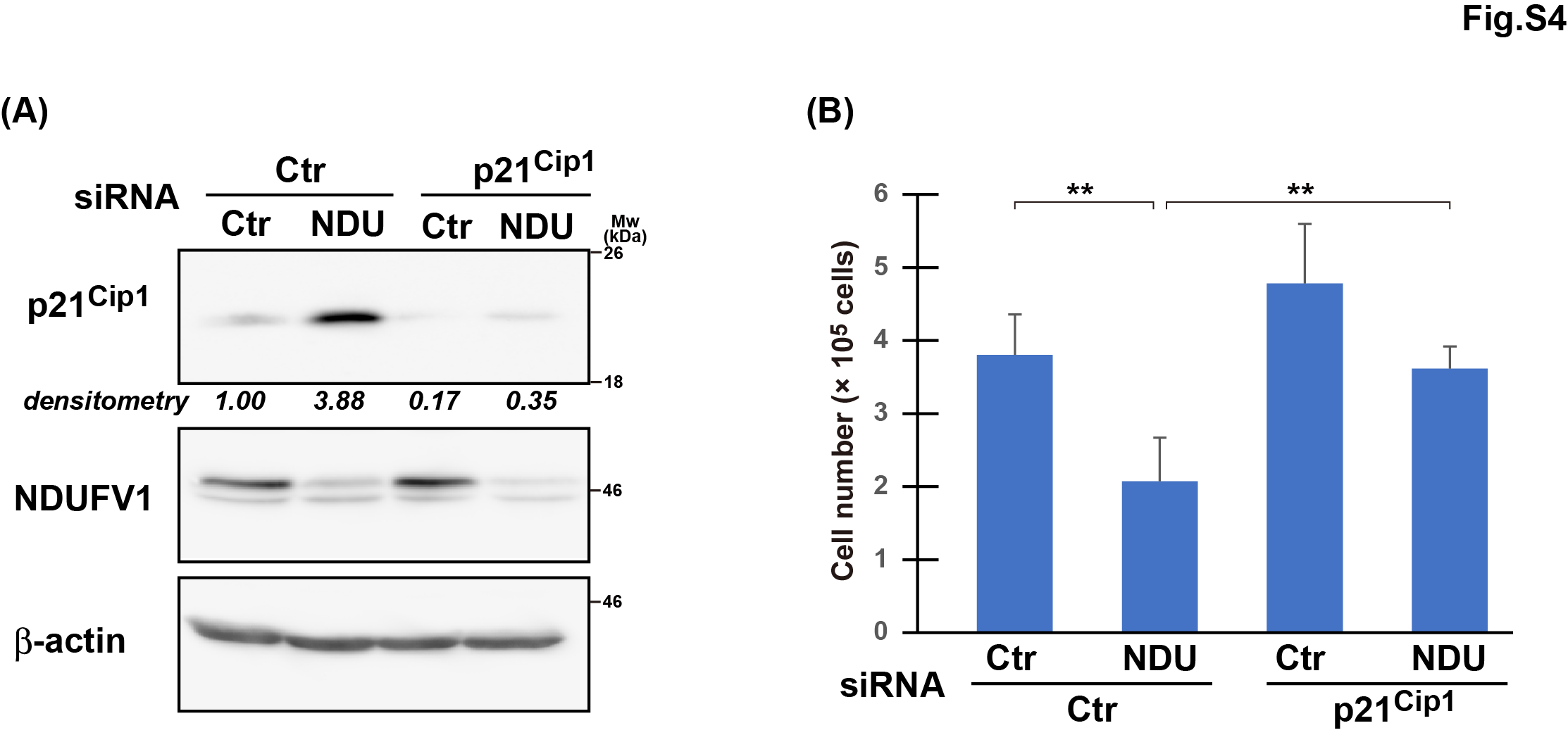

Supplement: Supplementary file 4 — Fig. S4. p21 Cip1 induction mediates cell proliferation arrest under NDUFV1 knockdown in MDA‐MB‐231 cells. (A) Western blotting with the indicated antibodies using MDA‐MB‐231 cells after treatment with siRNAs against NDUFV1 (NDU), p21 Cip1 , negative control siRNA (Ctr) or combinations as indicated for 72 h. β‐actin is the loading control. (B) Cell proliferation and viability were assessed after transfection with siRNAs for 72 h. **P < 0.01. [file MOL2-19-1775-s004.tif]

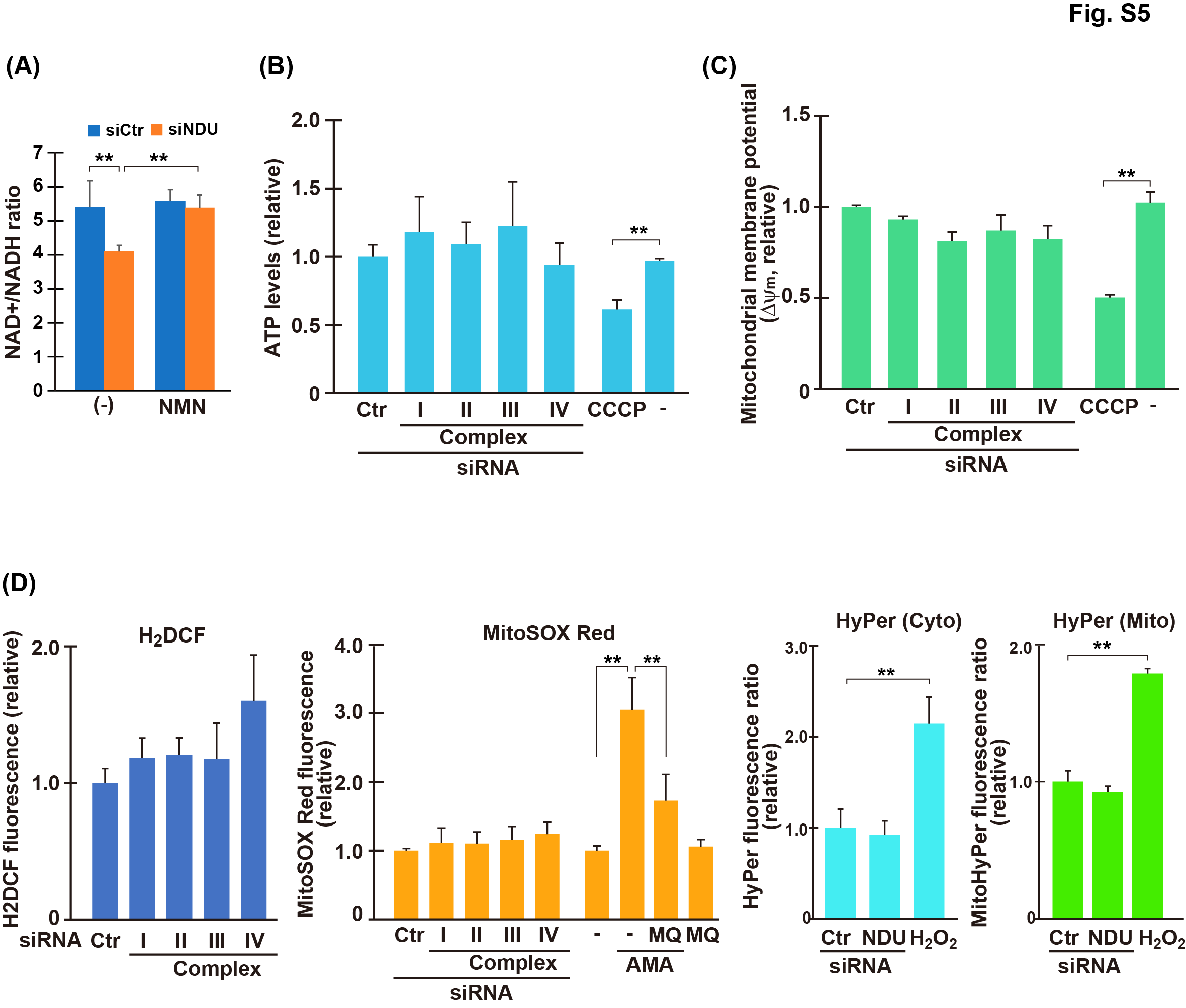

Supplement: Supplementary file 5 — Fig. S5. Effect of NDUFV1 and other ETC component knockdown on mitochondrial membrane potential, NAD+/NADH, ATP, and ROS levels. (A) MDA‐MB‐231 cells were treated with siRNA (Ctr, control; NDU, NDUFV1) in the presence or absence of 1 mm β‐nicotinamide mononucleotide (NMN). The NAD+/NADH ratio was evaluated after 24 h. (B, C) ATP levels (B) and ΔΨm (C) were examined in MDA‐MB‐231 cells using an ATP determination kit and the Mito‐ID Membrane Potential Cytotoxicity kit, respectively, after transfection with siRNAs (I, NDUFV1; II, SDHA; III, UQCRFS1; IV, SURF1) for 48 h. Values were presented relative to the Ctr. Carbonyl cyanide 3‐chlorophenylhydrazone (CCCP, FUJIFILM Wako Pure Chemical Corporation) was used as a control to induce mitochondrial depolarization. (D) Intracellular ROS levels were analyzed with 2′, 7′‐dichlorodihydrofluorescein diacetate (H2DCFDA) and MitoSOX Red using flow cytometry after transfection with the indicated siRNA for 48 h. The mean fluorescence intensity of H2DCFDA and MitoSOX Red, obtained from at least 10 000 cells, was presented as a ratio to the Ctr. Antimycin A (AMA, 10 μm) and mitochondria‐specific antioxidant, 10‐(6′‐ubiquinolyl) decyltriphenylphosphonium bromide (MQ, 0.5 μm) were used to modulate the ROS levels in the mitochondria. To measure the HyPer response, cells were infected with retroviral constructs expressing HyPer (Cyto or Mito, materials and methods). After transfection with the indicated siRNA for 48 h, images were captured using a CQ1 confocal quantitative image cytometer and analyzed using Cell Pathfinder software. After subtracting background, the HyPer response was evaluated as the fluorescence ratio (ex 488/ex 405). The values are presented relative to the Ctr. H2O2 treatment (50 μm, 10 min) was employed as a positive control. **P < 0.01. [file MOL2-19-1775-s008.tif]

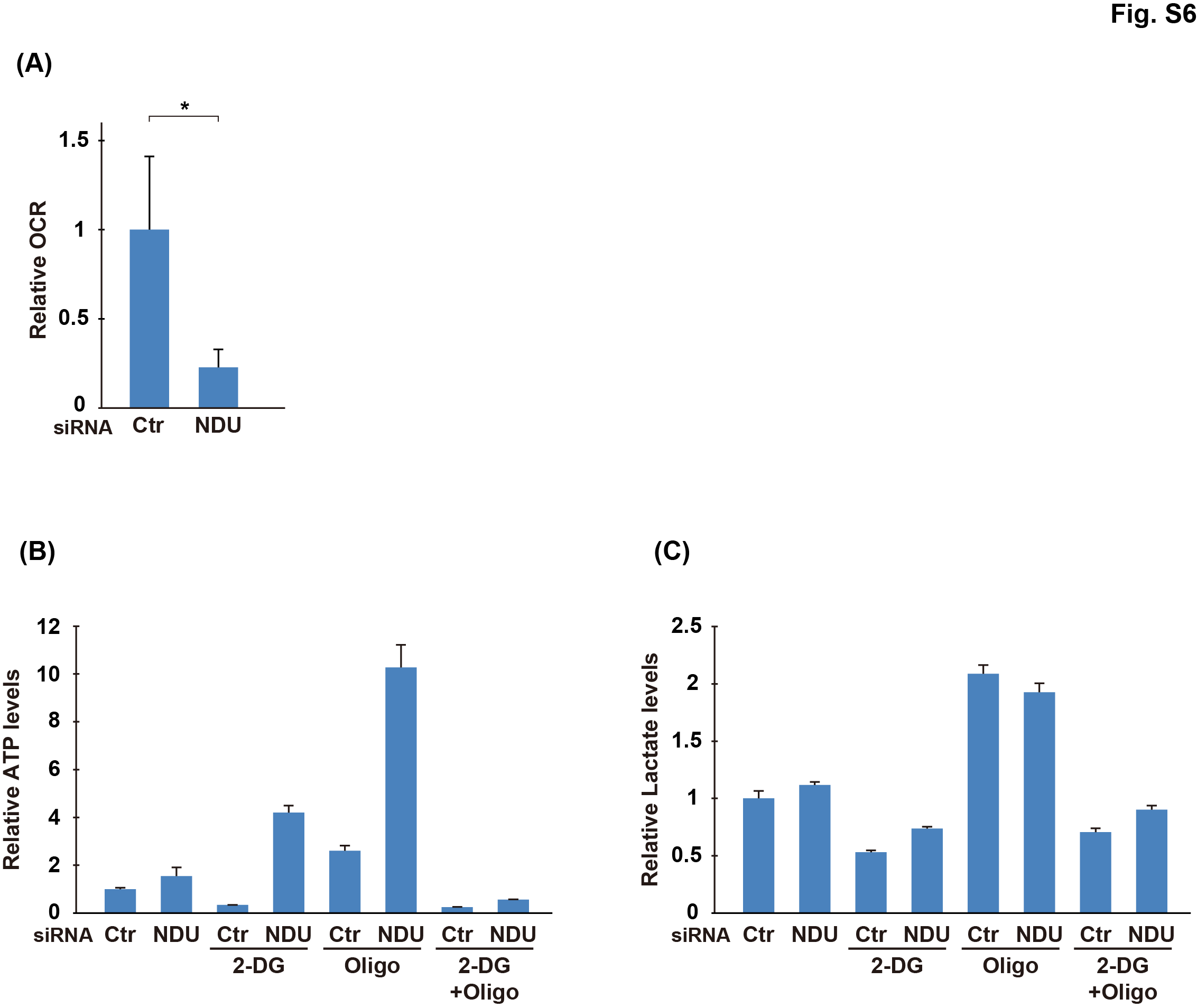

Supplement: Supplementary file 6 — Fig. S6. NDUFV1 knockdown shifts energy metabolism to glycolysis. (A) The oxygen consumption rate (OCR) was examined in MCF‐7 cells using the Extracellular OCR Plate Assay Kit after transfection with siRNA (Ctr, control; NDU, NDUFV1) for 48 h. Values are relative to control (Ctr). (B, C) ATP and (B) lactate (C) levels were examined in MCF‐7 cells using an ATP determination kit and Lactate Assay Kit‐WST, respectively. MCF‐7 cells were transfected with siRNA (Ctr, control; NDU, NDUFV1) for 48 h and then treated with 22.5 mm 2‐deoxy‐d‐glucose (2‐DG) or 1.25 μm oligomycin (Oligo) or both at 37 °C for 5 h. The cell lysate and the culture supernatants were used for ATP (B) and lactate (C) assays, respectively. Values are relative to control (Ctr, no treatment). [file MOL2-19-1775-s019.tif]

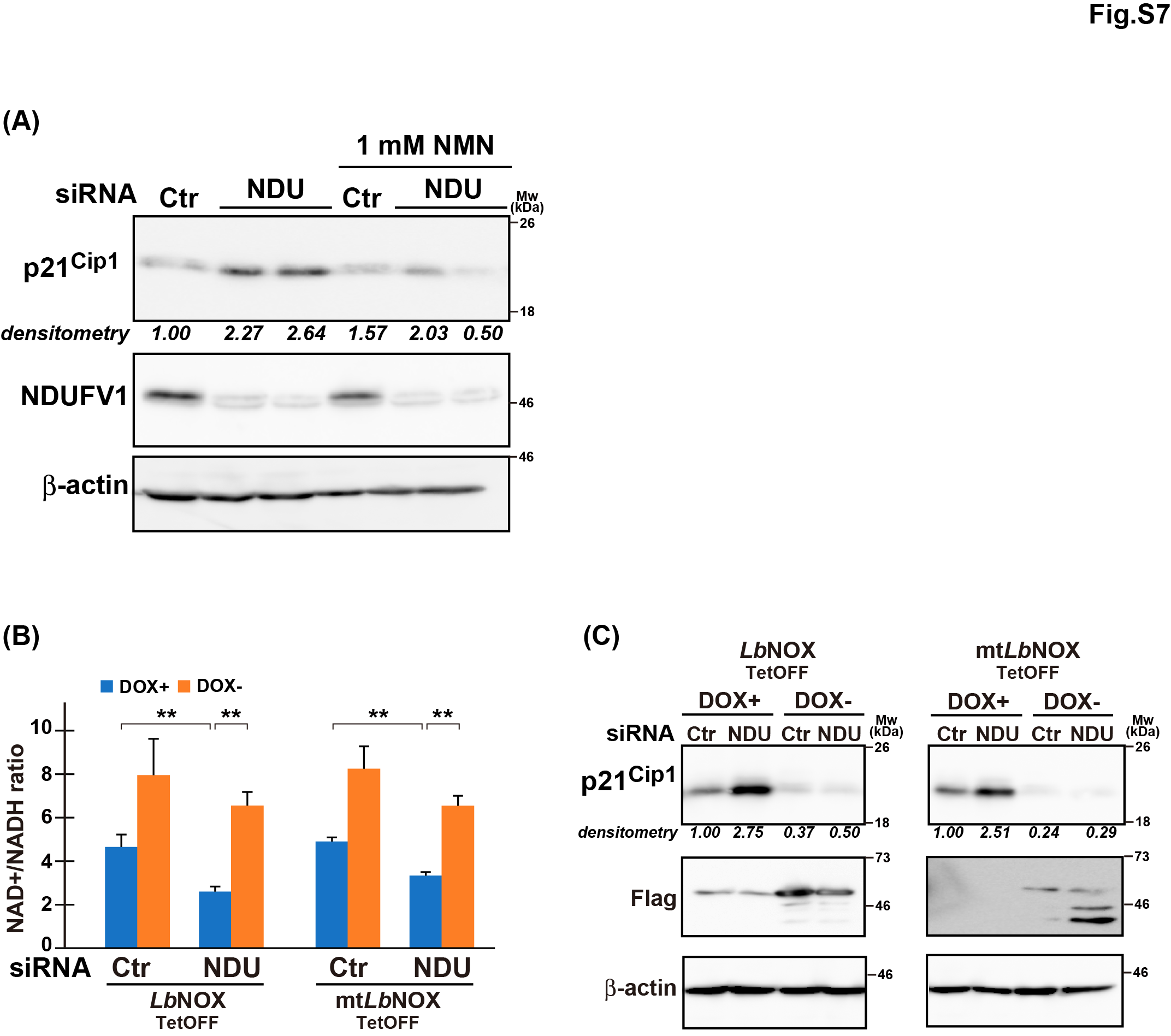

Supplement: Supplementary file 7 — Fig. S7. Decrease in the NAD+/NADH ratio signals to upregulate p21 Cip1 expression in MDA‐MB‐231 cells. (A) Western blotting with the indicated antibodies in MDA‐MB‐231 cells after transfection with siRNAs against NDUFV1 (NDU) or negative control siRNA (Ctr) for 72 h with or without 1 mm β‐nicotinamide mononucleotide (NMN). β‐actin is the loading control. (B, C) MDA‐MB‐231 cells were infected with doxycycline (DOX)‐responsive lentivirus constructs (TetOFF) expressing Flag‐tagged native (LbNOX), mitochondrial‐targeted LbNOX (mitoLbNOX). The NAD+/NADH ratio was determined after siRNA treatment for 24 h in the presence (+) or absence (−) of DOX (2 ng·mL−1) (B). Western blotting was performed 72 h after siRNA transfection with (+) or without (−) DOX (C). β‐actin is the loading control. **P < 0.01. [file MOL2-19-1775-s002.tif]

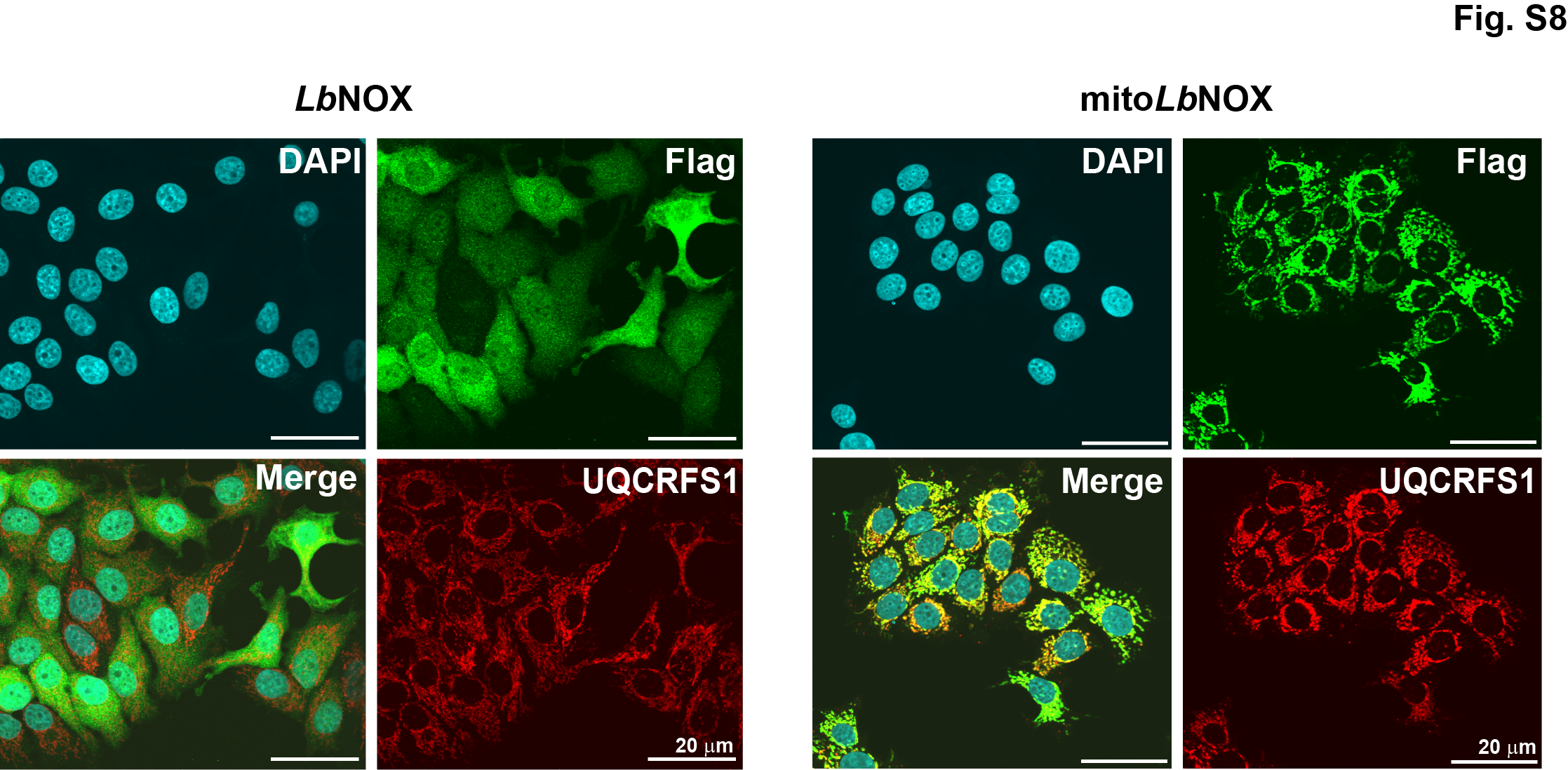

Supplement: Supplementary file 8 — Fig. S8. The subcellular localization of LbNOX and mitoLbNOX. The subcellular localization of LbNOX and mitoLbNOX was examined by immunocytochemistry using antibodies against Flag and UQCRFS1 after seeding LbNOX‐ or mitoLbNOX‐expressing MCF7 cells for 24 h with DOX (0.5 μg·mL−1). The cell nuclei were labeled with DAPI. Scale bar: 20 μm. [file MOL2-19-1775-s017.tif]

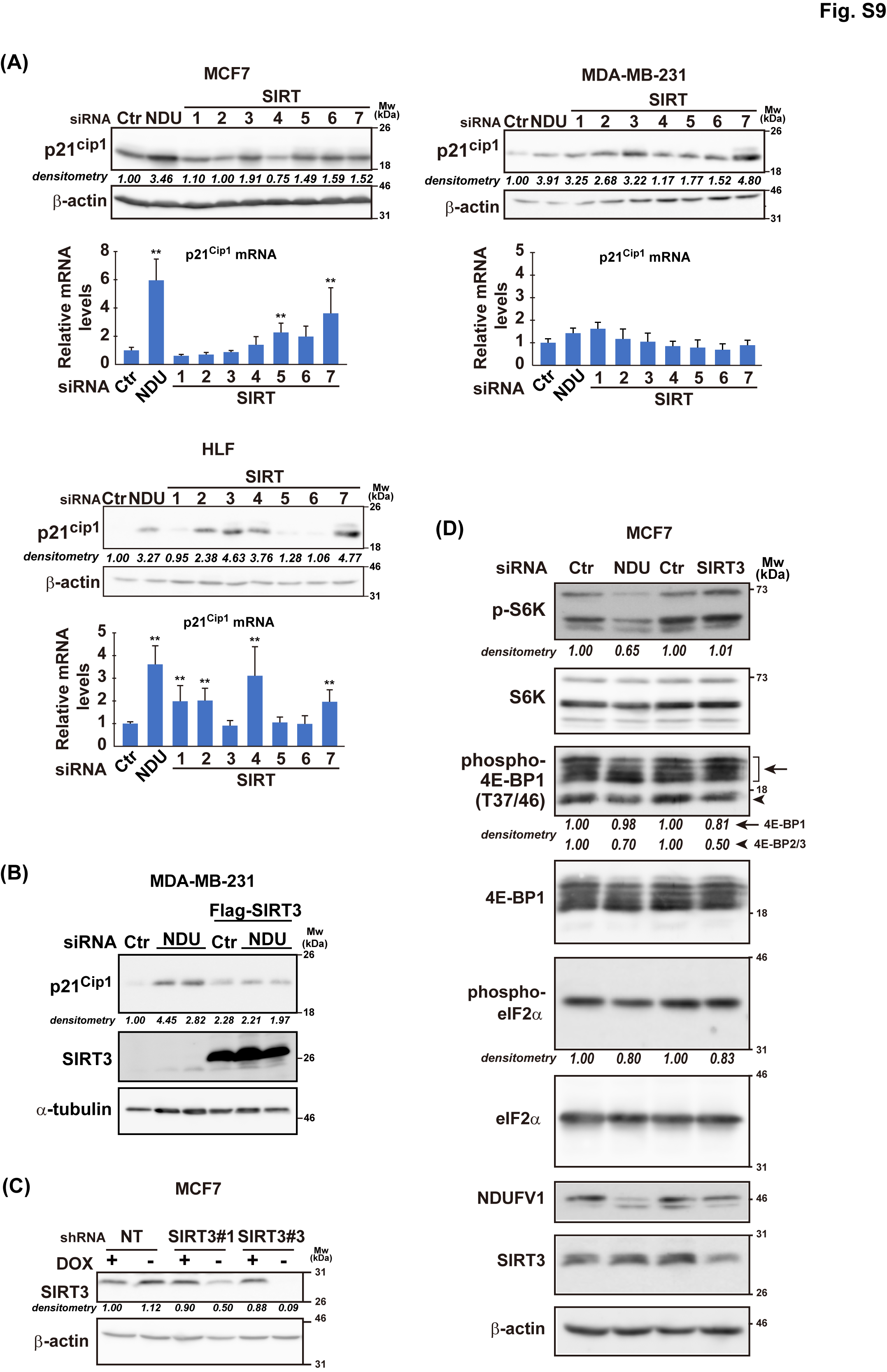

Supplement: Supplementary file 9 — Fig. S9. Effect of SIRTs knockdown on p21 Cip1 protein and mRNA levels, and translational machinery. (A) The p21 Cip1 protein and mRNA levels were examined by western blotting and qRT‐PCR, respectively, after transfection with the indicated siRNA for 48 or 72 h. Silencing efficiencies of siRNAs are indicated in Table S5. β‐actin is the loading control. The band intensity values and the mRNA levels are shown relative to the Ctr. **P < 0.01. (B) MDA‐MB‐231 cells expressing Flag‐tagged SIRT3 (Flag‐SIRT3) or control (mock) were treated with NDUFV1 (NDU) siRNA or negative control siRNA (Ctr). After 72 h, the protein levels of p21 Cip1 were examined by western blotting. α‐Tubulin is the loading control. (C) MCF‐7 cells expressing doxycycline (DOX)‐responsive (TetOFF) SIRT3#1, SIRT3#3, or non‐target control (NT) shRNA were cultured for 72 h with (+) or without (−) DOX (1 μg·mL−1). Knockdown of SIRT3 was examined by western blotting. β‐Actin is the loading control. (D) MCF‐7 cells were incubated with siRNAs against NDUFV1 (NDU), SIRT3, or negative control (Ctr) for 48 h. Western blotting with the indicated antibodies. Band intensities measured with ImageJ are shown as relative to the control (Ctr). β‐Actin is the loading control. An arrow and an arrowhead indicate the phosphorylated forms of 4E‐BP1 and 4E‐BP2/3, respectively. [file MOL2-19-1775-s018.tif]

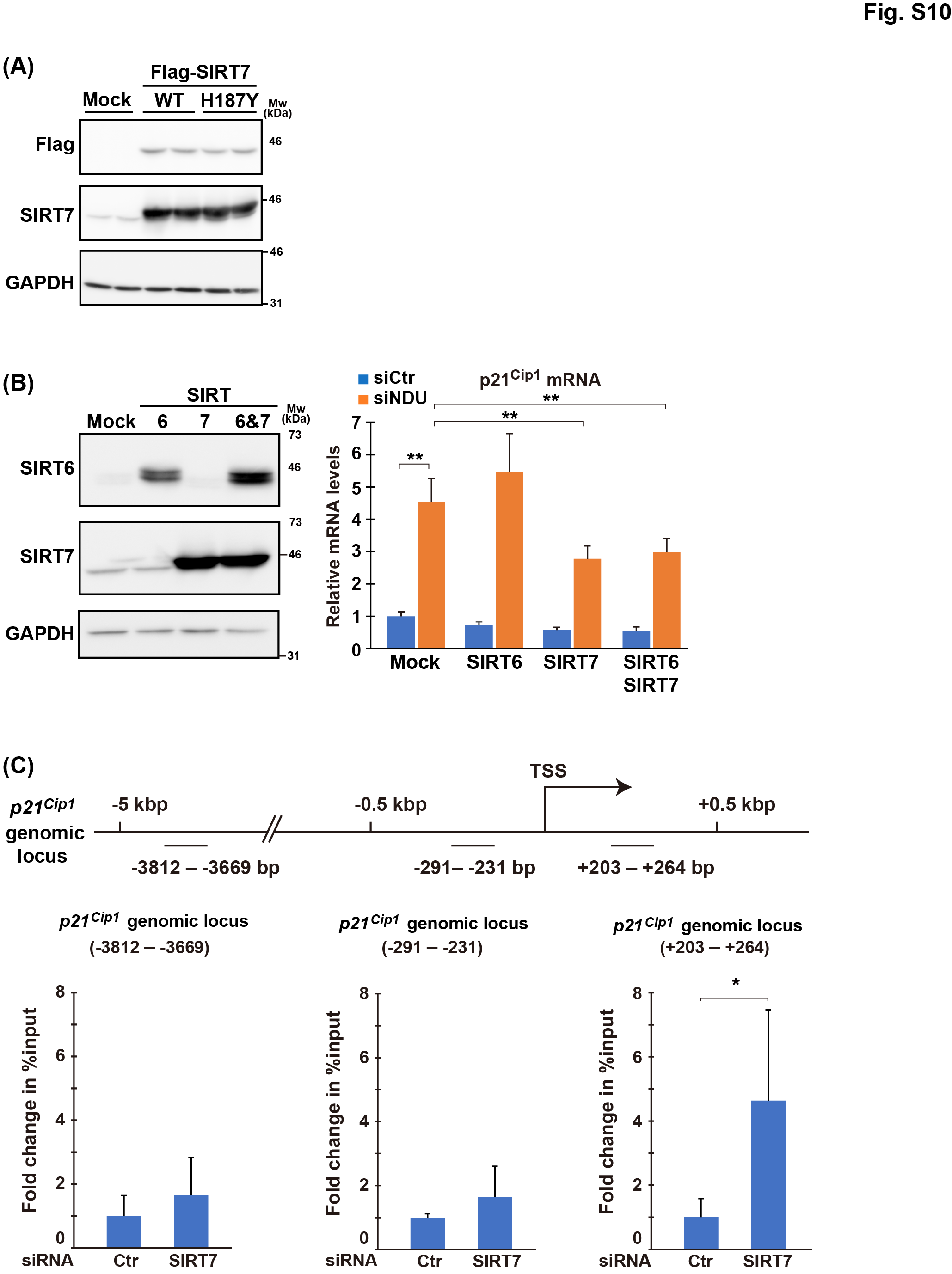

Supplement: Supplementary file 10 — Fig. S10. Effect of SIRT7 and SIRT6 overexpression on p21 Cip1 expression. (A) MCF7 cells were infected with lentiviral constructs expressing Flag‐tagged SIRT7 WT, SIRT7 H187Y (Materials and methods), or control (Mock). After selection, exogenous SIRT7 expression was confirmed by western blotting with the indicated antibodies. GAPDH was the loading control. (B) Cells expressing Flag‐tagged SIRT7 WT or control (Mock) were infected with retroviral vectors (Mock or Flag‐tagged SIRT6). After selection, exogenous SIRT6 and SIRT7 expression was confirmed as above. GAPDH was the loading control. p21 Cip1 mRNA expression was examined by qRT‐PCR after transfection with the indicated siRNA for 48 h. Values are presented relative to the control (Mock/Ctr). (C) CUT&RUN was performed to examine the enrichment of acetylated H3K18 on the p21 Cip1 genomic locus after 24 h of siRNA transfection (materials and methods). The fold changes normalized to the Ctr were plotted. TSS; transcription start site. *P < 0.05, **P < 0.01. [file MOL2-19-1775-s016.tif]

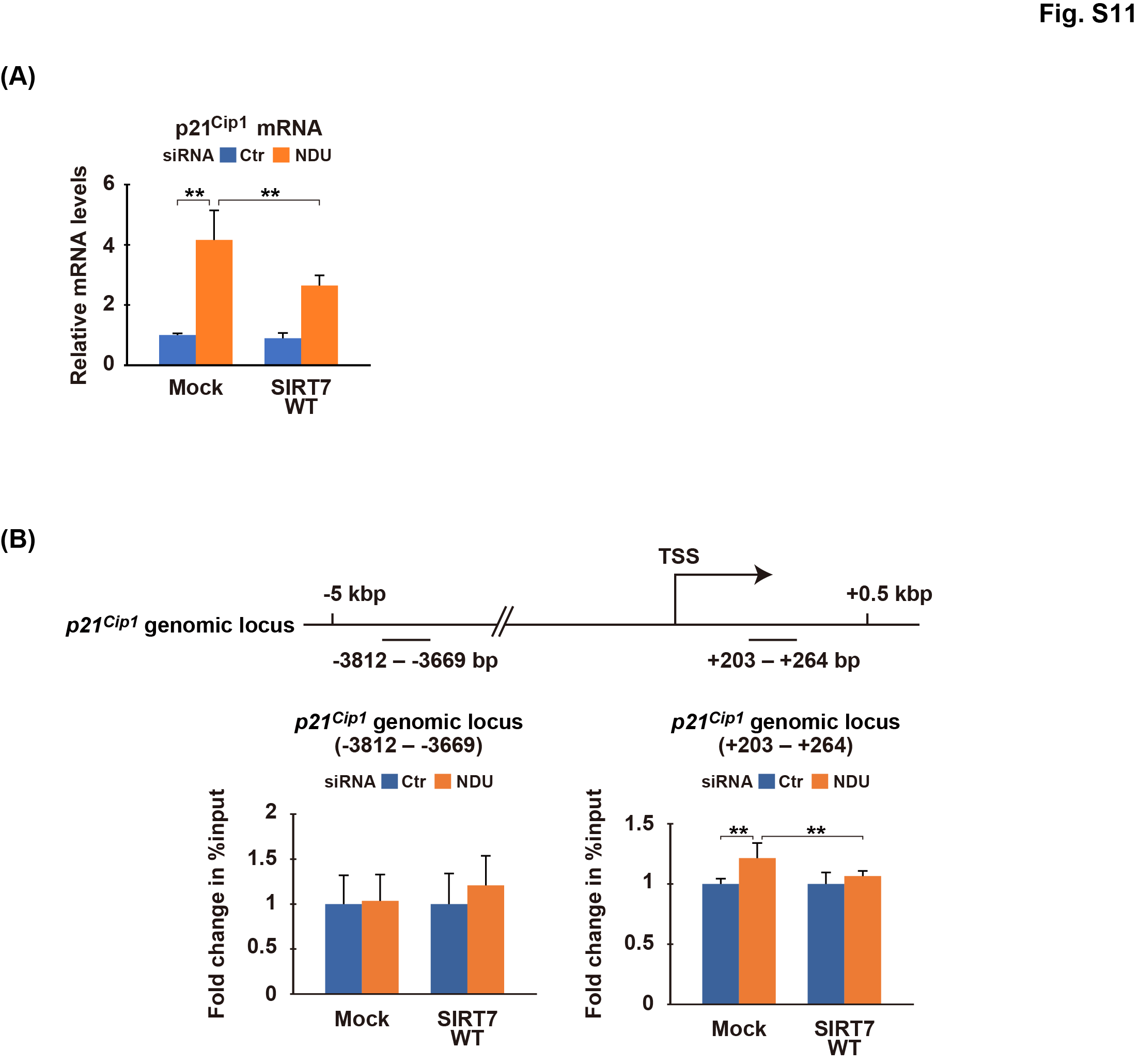

Supplement: Supplementary file 11 — Fig. S11. Transcription of p21 Cip1 is suppressed by SIRT7 under CI activity in HLF cells. (A, B) HLF cells expressing Flag‐tagged wild‐type SIRT7 (SIRT7 WT) or control (mock) were treated with NDUFV1 (NDU) siRNA or negative control siRNA (Ctr). After 48 h, the mRNA levels of p21 Cip1 were examined by qRT‐PCR (A). Values are relative to control (mock/Ctr). In (B), ChIP was performed to examine the enrichment of acetyl H3K18 on the p21 Cip1 locus after siRNA treatment for 54 h. The precipitated DNA was analyzed by qPCR using primers specific to the p21 Cip1 locus (Table S4), as indicated in the map. The fold changes were normalized to the Ctr. TSS; transcription start site. **P < 0.01. [file MOL2-19-1775-s014.tif]

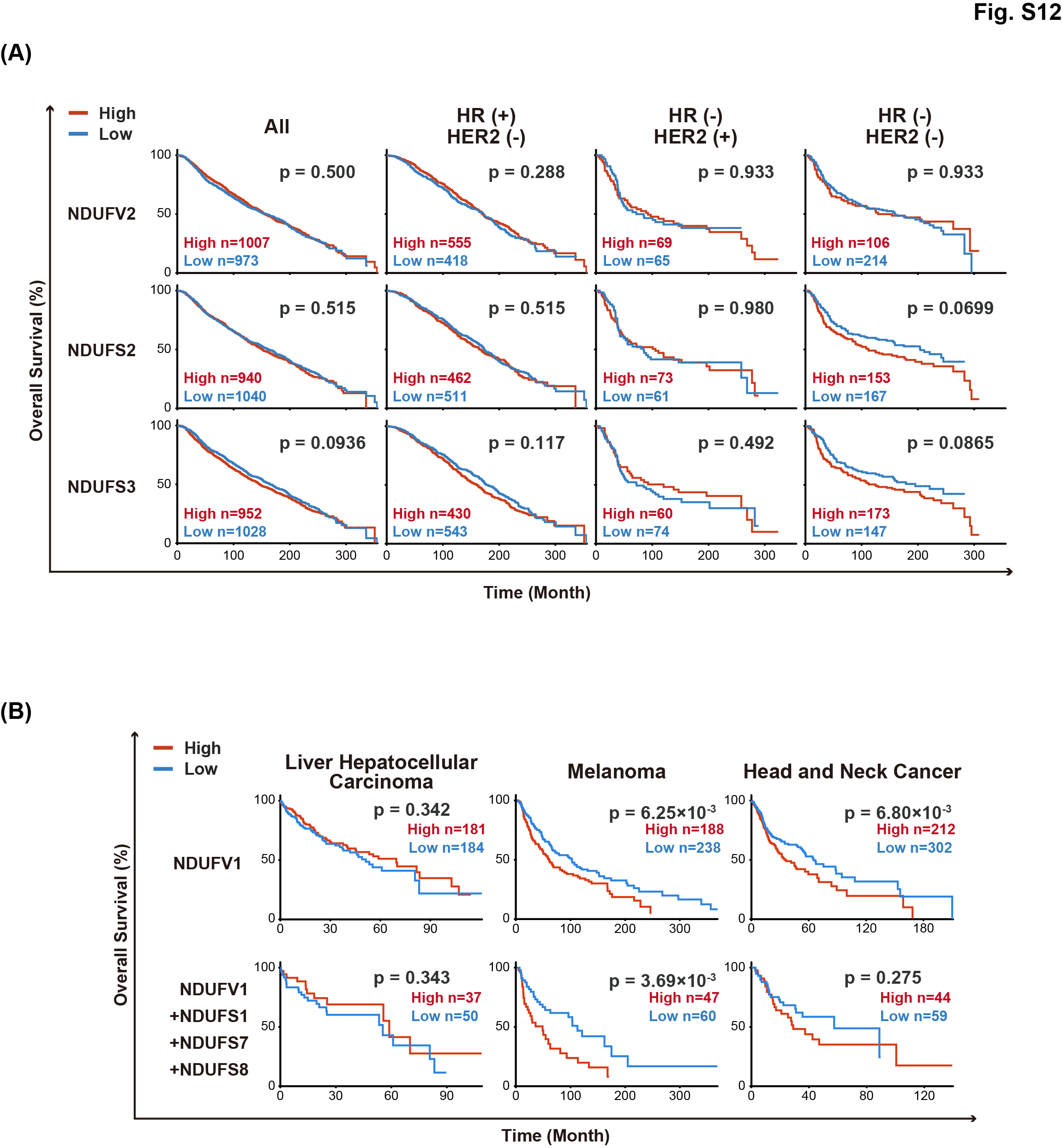

Supplement: Supplementary file 12 — Fig. S12. The impact of expression levels of CI subunits on overall survival in patients with breast cancer and other cancers. (A) Kaplan–Meier plots of the overall survival of patients with each subtype of breast cancer, categorized by the expression levels [high, z‐score >0; low, z‐score ≤0] of the indicated subunits, were obtained as in Fig. 6. (B) Kaplan–Meier plots with patient categorized by expression levels [High, z‐score >0; Low, z‐score ≤ 0] of the indicated subunits were obtained in each cancer type using the TCGA PanCancer Atlas dataset through cBioPortal (described in Materials and methods). n, number of samples. [file MOL2-19-1775-s001.tif]

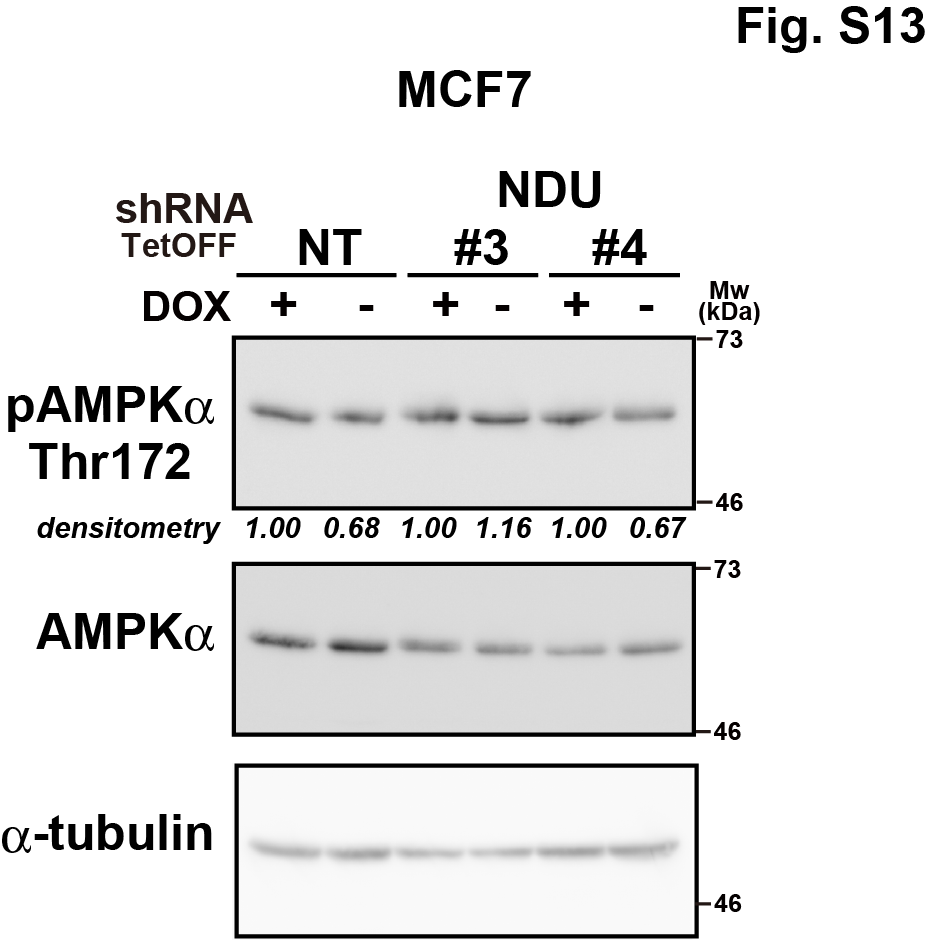

Supplement: Supplementary file 13 — Fig. S13. NAD+‐SIRT‐p21Cip1 pathway is independent of AMPK. Doxycycline (DOX)‐responsive (TetOFF) shRNA [NT, non‐target control; NDU, NDFUV1]‐expressing MCF7 cells were incubated for 72 h in the presence (+) or absence (−) of DOX (1.0 μg·mL−1). Western blotting was performed with the indicated antibodies. The loading control is α‐tubulin. The band intensities measured with ImageJ are shown relative to the control (NT/DOX+) after normalization using loading control. [file MOL2-19-1775-s007.tif]
