# Supplementary material for: Respiratory complex I‐mediated NAD + regeneration regulates cancer cell proliferation through the transcriptional and translational control of p21 Cip1 expression by SIRT3 and SIRT7
Source: Mol Oncol. 2025 Jan 28;19(6):1775–96. doi: 10.1002/1878-0261.13808 (PMC12161471; doi:10.1002/1878-0261.13808)
Supplement: Supplementary file 14 — Table S1. Cell lines used in the study. [file MOL2-19-1775-s013.pdf]

Table S1. Cell lines used in the study

Cell line	Cellosaurus	Cell bank (Catalog#)	Culture medium	p21Cip1 [*9] mRNA [*10]	p21Cip1 [*9] Protein [*11]
MCF7	CVCL_0031	ATCC (HTB-22)	DMEM / Low glucose [*1]<br>10% FBS	+	+
T47D	CVCL_0553	ATCC (HTB-133)	DMEM / Low glucose [*1]<br>10% FBS	*n.s.*	*n.s.*
MDA-MB-231	CVCL_0062	ATCC (HTB-26)	DMEM / High glucose [*2]<br>10% FBS	*n.s.*	+
MDA-MB-436	CVCL_0623	ATCC (HTB-130)	DMEM / High glucose [*2]<br>10% FBS	*n.s.*	*n.s.*
BT-549	CVCL_1092	ATCC (HTB-122)	RPMI [*3]<br>10% FBS	+	+
MDA-MB-361	CVCL_0620	ATCC (HTB-27)	DMEM / High glucose [*2]<br>10% FBS	*n.s.*	*n.s.*
SKBR3	CVCL_0033	ATCC (HTB-30)	McCoy's 5A [*4]<br>10% FBS	*n.s.*	*n.s.*
HCC1954	CVCL_0030	ATCC (CRL-2338)	RPMI [*3]<br>10% FBS	*n.s.*	*n.s.*
TertHMECs			Ref)<br>*Cell Death Dis.* 2015<br>6(1):e1619. [*5]	+	+
HMLER			Ref)<br>*FEBS J.* 2019<br>286(3):459-478. [*5]	+	+
HLF	CVCL_2255	JCRB (JCRB0405)	DMEM / Low glucose [*1]<br>5% FBS	+	+
JHH-1	CVCL_2785	JCRB (JCRB1029)	Williams'E medium [*6]<br>10% FBS	*n.s.*	*n.s.*
JHH-2	CVCL_2786	JCRB (JCRB1028)	Williams'E medium [*6]<br>10% FBS	*n.s.*	+
JHH-4	CVCL_2787	JCRB (JCRB0435)	MEM [*7]<br>10% FBS	*n.s.*	N.D.
JHH-6	CVCL_2788	JCRB (JCRB1030)	Williams'E medium [*6]<br>10% FBS	N.D.	+
HepG2	CVCL_0027	JCRB (JCRB1054)	DMEM / Low glucose [*1]<br>10% FBS	*n.s.*	*n.s.*
Hc3716 -h*TERT*	CVCL_B6EH		Hepatocyte Culture Medium [*8]<br>2% DMSO, 5% FBS<br>10% human serum<br>Ref) *Cancer Sci.* 2010<br>101:1678-1685	+	+

*1, D-Glucose 1.0 g/L (5.55 mM), Sodium Pyruvate, 110 mg/L (1 mM)
*2, D-Glucose 4.5 g/L (25 mM), Sodium Pyruvate 110 mg/L (1 mM)
*3, D-Glucose 2.0 g/L (11.11 mM), Sodium Pyruvate 0 mg/L (0 mM)
*4, D-Glucose 3.0 g/L (16.66 mM), Sodium Pyruvate 0 mg/L (0 mM)
*5, D-Glucose 1.44 g/L (8 mM), Sodium Pyruvate 110 mg/L (1 mM)
*6, D-Glucose 2.0 g/L (11.11 mM), Sodium Pyruvate 25 mg/L (0.23 mM)
*7, D-Glucose 1.0 g/L (5.55 mM), Sodium Pyruvate 0 mg/L (0 mM)
*8, D-Glucose 2.0 g/L (11.11 mM), Sodium Pyruvate 25 mg/L (0.23 mM)
*9, +, Upregulated
*10, See Fig. S2
*11, See Fig. S3

N.D., not determined; *n.s.*, not significant
