# Supplementary material for: Respiratory complex I‐mediated NAD + regeneration regulates cancer cell proliferation through the transcriptional and translational control of p21 Cip1 expression by SIRT3 and SIRT7
Source: Mol Oncol. 2025 Jan 28;19(6):1775–96. doi: 10.1002/1878-0261.13808 (PMC12161471; doi:10.1002/1878-0261.13808)
Supplement: Supplementary file 15 — Table S2. List of targeting sequences for shRNA. [file MOL2-19-1775-s015.pdf]

Table S2. List of targeting sequences for shRNA

shRNA	Target sequence
shNT	CAA CAA GAT GAA GAG CAC CAA
shNDUFV1#1	CGA GAT CAA GAC ATC GGG TTT
shNDUFV1#2	CTG AAG GAT GAA GAC CGG ATT
shNDUFV1#3	CCG CTC GAC GGA CAT CGT GAA
shNDUFV1#4	CCG GGA GAT CTT ACG CCA TGA
shSIRT3#1	GCG GCT CTA CAC GCA GAA CAT
shSIRT3#3	GTG GGT GCT TCA AGT GTT GTT
