# Supplementary material for: Respiratory complex I‐mediated NAD + regeneration regulates cancer cell proliferation through the transcriptional and translational control of p21 Cip1 expression by SIRT3 and SIRT7
Source: Mol Oncol. 2025 Jan 28;19(6):1775–96. doi: 10.1002/1878-0261.13808 (PMC12161471; doi:10.1002/1878-0261.13808)
Supplement: Supplementary file 16 — Table S3. List of antibodies. [file MOL2-19-1775-s012.pdf]

Table S3. List of antibodies

Antibodies	Manufacture	Catalog number	Source
NDUFV1	Proteintech	11238-1-AP	Rabbit Polyclonal Antibody
SDHA	Thermo Fisher Scientific	459200	Mouse Monoclonal Antibody
UQCRFS1	Proteintech	18443-1-AP	Rabbit Polyclonal antibody
SURF1	Proteintech	15379-1-AP	Rabbit Polyclonal antibody
$p21^{Cip1}$	Cell Signaling Technology	2947	Rabbit Monoclonal Antibody
P27KIP1	BD Biosciences	554069	Mouse Monoclonal Antibody
SOD2 (acetyl K68)	Abcam	ab137037	Rabbit Monoclonal Antibody
SOD2	Cell Signaling Technology	13141	Rabbit Monoclonal Antibody
SIRT3	Cell Signaling Technology	5490	Rabbit Monoclonal Antibody
SIRT7	Cell Signaling Technology	5360	Rabbit Monoclonal Antibody
Flag	Merck KGaA	A2220	Mouse Monoclonal Antibody
AMPKα	Cell Signaling Technology	2532	Rabbit Polyclonal antibody
AMPKα (Phospho-Thr172)	Cell Signaling Technology	2535	Rabbit Monoclonal Antibody
SLC1A3	Novus Biologicals	NB100-1869	Rabbit Polyclonal antibody
S6K	Cell Signaling Technology	9202	Rabbit Polyclonal antibody
S6K (Phospho-Thr389)	Cell Signaling Technology	9205	Rabbit Polyclonal antibody
4E-BP1	Cell Signaling Technology	9644	Rabbit Monoclonal Antibody
4E-BP1 (Phospho-Thr37/46)	Cell Signaling Technology	2855	Rabbit Monoclonal Antibody
eIF2α	Cell Signaling Technology	5324	Rabbit Monoclonal Antibody
eIF2α (Phospho-Ser51)	Cell Signaling Technology	3398	Rabbit Monoclonal Antibody
α-tubulin	Thermo Fisher Scientific	13-8000	Mouse Monoclonal Antibody
β-actin	Santa Cruz Biotechnology	sc-47778	Mouse Monoclonal Antibody
GAPDH	Merck KGaA	MAB374	Mouse Monoclonal Antibody
Mouse IgG HRP-linked antibody	Cytiva	NA931	
Rabbit IgG HRP-linked antibody	Cytiva	NA934	
Streptavidin-HRP conjugates	Cytiva	RPN1231	
Anti-Mouse IgG, Alexa Fluor 488	Thermo Fisher Scientific	A28175	
Anti-Mouse IgG, Alexa Fluor 568	Agilent	A11011	
Rabbit immunoglobulin (normal)	Agilent	X090310-8	
