# Supplementary material for: Respiratory complex I‐mediated NAD + regeneration regulates cancer cell proliferation through the transcriptional and translational control of p21 Cip1 expression by SIRT3 and SIRT7
Source: Mol Oncol. 2025 Jan 28;19(6):1775–96. doi: 10.1002/1878-0261.13808 (PMC12161471; doi:10.1002/1878-0261.13808)
Supplement: Supplementary file 17 — Table S4. Primer sequences for qPCR, ChIP, and CUT&RUN. [file MOL2-19-1775-s003.pdf]

Table S4. Primer sequences for qPCR, ChIP, and CUT&RUN

Primer sequences for qPCR

Gene	Forward (5' → 3')	Reverse (5' → 3')
p21^{Cip1}	AGCAGAGGAAGACCATGTGGAC	TTTCGACCCTGAGAGTCTCCAG
NDUFV1<br>Complex I	CCATCCGAGAGGCCTATGAG	CATAGCCAGAGCCACAAGCA
SDHA<br>Complex II	GGCATTCCCACCAACTACAAG	CACAATCTGATCCTGGCCATT
UQCRC1<br>Complex III	CTTGCTGGGTGAACACTTTG	GGACGAACATCATGTCATCGT
SURF1<br>Complex IV	GGGTTCGTTCCCAGGAAGA	CCCTCAATCTGGCCTTTCTG
SIRT1	TGCGGGAATCCAAAGGATAA	CAGGCAAGATGCTGTTGCA
SIRT2	CAAGAAACATCCGGAACCCTTC	AGATGGTTGGCTTGAACTGC
SIRT3	TCTGCACCGGCGTTGTG	CAGCGGCTCCCCAAAGA
SIRT4	TGCAAGAGCGTTTCCAAGTC	TCCACATTGAACGCAGGTTG
SIRT5	CCCAGAACATCGATGAGC	GCCACAACTCCACAAGAGG
SIRT6	GCTGACCTCCGCATCCAT	AGCCCCAGGTGCTTCATG
SIRT7	ACACCATTGTGCACTTTGGG	AGAGGCGTGGGTACTTCTTTAG
TBP<br>TATA-binding protein	GCCAGGCACCACAGCTCTT	CGGCAAGGGTGCAGTTG

Primer sequences for ChIP and CUT&RUN

Position of the human *p21^{Cip1}* genomic locus	Forward (5' → 3')	Reverse (5' → 3')
-3812 – -3669	TCAATGCCACCACCTTAACA	AGAGAGGCATCCTCCAGACA
-291 – -231	TCCTGGAGAGTGCCAACTCAT	CACGAAGTGAGCCACAAATCTG
+203 – +264	CGCGAGGATGCGTGTTC	CATTCACCTGCCGCAGAAA
