# Supplementary material for: Respiratory complex I‐mediated NAD + regeneration regulates cancer cell proliferation through the transcriptional and translational control of p21 Cip1 expression by SIRT3 and SIRT7
Source: Mol Oncol. 2025 Jan 28;19(6):1775–96. doi: 10.1002/1878-0261.13808 (PMC12161471; doi:10.1002/1878-0261.13808)
Supplement: Supplementary file 18 — Table S5. Silencing efficiency of siRNAs. [file MOL2-19-1775-s005.pdf]

Table S5. Silencing efficiency of siRNAs

Cell line \ siRNA	Complex I	Complex II	Complex III	Complex IV (%)
MCF7	77.7	92.6	86.7	83.9
MDA-MB-231	80.1	92.2	87.7	83.3
HLF	90.0			
JHH-4	93.6			

Cell line \ siRNA	SIRT 1	SIRT 2	SIRT 3	SIRT 4	SIRT 5	SIRT 6	SIRT 7 (%)
MCF7	92.9	92.3	84.1	47.6	85.3	70.1	82.9
MDA-MB-231	89.2	90.8	85.6	52.0	92.1	37.8	85.7
HLF	87.9	92.8	53.7	48.6	84.3	48.3	94.3

After performing qRT-PCR, the silencing efficiency of siRNAs is measured by the percentage of target mRNA reduction in siRNA-transfected cells relative to control siRNA-transfected cells.
