# Supplementary material for: Respiratory complex I‐mediated NAD + regeneration regulates cancer cell proliferation through the transcriptional and translational control of p21 Cip1 expression by SIRT3 and SIRT7
Source: Mol Oncol. 2025 Jan 28;19(6):1775–96. doi: 10.1002/1878-0261.13808 (PMC12161471; doi:10.1002/1878-0261.13808)
Supplement: Supplementary file 19 — Table S6. Quantification of aspartate and TCA cycle intermediates in MCF7 cells. [file MOL2-19-1775-s009.pdf]

Table S6. Quantification of aspartate and TCA cycle intermediates in MCF7 cells

Cell: MCF7

	**Relative Area**					
	**siControl**		**siNDUFV1**		**Ratio**	
	Mean	S.D.	Mean	S.D.	**siNDU / siCtr**	*p*-value
Asparatate	0.598	0.046	1.048	0.049	1.752	0.011
α-ketoglutarate	0.111	0.003	0.070	0.006	0.629	0.013
Citrate	0.361	0.010	0.342	0.006	0.947	0.153
Fumarate	0.008	0.001	N.D.	N.D.	<1	N.A.
Succinate	0.040	0.002	0.049	0.004	1.217	0.100

Details of the mass spectrometry analysis are described in the materials and methods.

The measurement data were processed with peak processing software [*Metabolomics*, 2010, 6:78-95]. The peak area of each metabolite was normalized to that of the appropriate internal standard. The values of relative area were then further normalized by the sample weight.

S.D., standard deviation; N.D., Not detected; N.A., Not available
